# Patterns of *Cis* Regulatory Variation in Diverse Human Populations

**DOI:** 10.1371/journal.pgen.1002639

**Published:** 2012-04-19

**Authors:** Barbara E. Stranger, Stephen B. Montgomery, Antigone S. Dimas, Leopold Parts, Oliver Stegle, Catherine E. Ingle, Magda Sekowska, George Davey Smith, David Evans, Maria Gutierrez-Arcelus, Alkes Price, Towfique Raj, James Nisbett, Alexandra C. Nica, Claude Beazley, Richard Durbin, Panos Deloukas, Emmanouil T. Dermitzakis

**Affiliations:** 1 Wellcome Trust Sanger Institute, Wellcome Trust Genome Campus, Hinxton, United Kingdom; 2 Department of Medicine, Division of Genetics, Brigham and Women's Hospital, Boston, Massachusetts, United States of America; 3 Program in Medical and Population Genetics, Broad Institute of Harvard and Massachusetts Institute of Technology, Cambridge, Massachusetts, United States of America; 4 Harvard Medical School, Boston, Massachusetts, United States of America; 5 Department of Genetic Medicine and Development, University of Geneva Medical School, Geneva, Switzerland; 6 Institute of Genetics and Genomics in Geneva (iGE3), Geneva, Switzerland; 7 Wellcome Trust Centre for Human Genetics, Oxford, United Kingdom; 8 Max Planck Institute Intelligent Systems and Max Planck Institute for Developmental Biology, Tübingen, Germany; 9 MRC CAiTE centre, School of Social and Community Medicine, University of Bristol, Bristol, United Kingdom; 10 Department of Epidemiology and Department of Biostatistics, Harvard School of Public Health, Boston, Massachusetts, United States of America; 11 Department of Neurology, Brigham and Women's Hospital, Boston, Massachusetts, United States of America; Stanford University School of Medicine, United States of America

## Abstract

The genetic basis of gene expression variation has long been studied with the aim to understand the landscape of regulatory variants, but also more recently to assist in the interpretation and elucidation of disease signals. To date, many studies have looked in specific tissues and population-based samples, but there has been limited assessment of the degree of inter-population variability in regulatory variation. We analyzed genome-wide gene expression in lymphoblastoid cell lines from a total of 726 individuals from 8 global populations from the HapMap3 project and correlated gene expression levels with HapMap3 SNPs located in *cis* to the genes. We describe the influence of ancestry on gene expression levels within and between these diverse human populations and uncover a non-negligible impact on global patterns of gene expression. We further dissect the specific functional pathways differentiated between populations. We also identify 5,691 expression quantitative trait loci (eQTLs) after controlling for both non-genetic factors and population admixture and observe that half of the *cis*-eQTLs are replicated in one or more of the populations. We highlight patterns of eQTL-sharing between populations, which are partially determined by population genetic relatedness, and discover significant sharing of eQTL effects between Asians, European-admixed, and African subpopulations. Specifically, we observe that both the effect size and the direction of effect for eQTLs are highly conserved across populations. We observe an increasing proximity of eQTLs toward the transcription start site as sharing of eQTLs among populations increases, highlighting that variants close to TSS have stronger effects and therefore are more likely to be detected across a wider panel of populations. Together these results offer a unique picture and resource of the degree of differentiation among human populations in functional regulatory variation and provide an estimate for the transferability of complex trait variants across populations.

## Author Summary

Variation among individuals in the degree to which genes are expressed (i.e. turned on or off) is a characteristic exhibited by all species, and studies have identified regions of the genome harboring genetic variation affecting gene expression levels. To assess the degree of human inter-population variability in regulatory variation, we describe mapping of regions of the genome that have functional effects on gene expression levels. We analyzed genome-wide gene expression in human cell lines derived from 726 unrelated individuals representing 8 global populations that have been genetically well-characterized by the International HapMap Project. We describe the influence of ancestry on gene expression levels within and between these diverse human populations and uncover a non-negligible impact on global patterns of gene expression. We identify ∼5,700 genes whose expression levels are associated with genetic variation located physically close to the gene, and we observe significant sharing of associations that is partially dependent on population genetic relatedness, among Asians, European-admixed, and African subpopulations. We identify biological functions affected by regulatory variation and describe common and unique characteristics of population-specific and population-shared associations. These results offer a unique picture and resource of the degree of differentiation among human populations in functional regulatory variation.

## Introduction

One of the fundamental questions of human population genetics is the extent to which human populations from around the world differ from one another. Population differentiation can be seen from the perspective of history, where neutral DNA sequence variation is used to reconstruct the relationships between populations given models of demography, migration, and genetic drift. The recent revolution of experimental approaches that address genome function has allowed the characterization of population differentiation with respect to variants that alter genome function. To date, two types of functional variants have attracted the most attention (i) variants that alter protein coding sequence (non-synonymous variants), and (ii) variants that are associated with levels of gene expression, i.e., regulatory variants that are also referred to as eQTLs (expression Quantitative Trait Loci). While there have been extensive studies of human population differentiation with respect to protein coding variants [1], [2], little is known about the degree of population differentiation of regulatory variants, either for those with regulatory effects on nearby genes (*cis*-eQTLs) or those acting over longer genomic distances (*trans*-eQTLs). This deficit of knowledge needs to be addressed given that it is likely that: (i) a large number of high-frequency eQTLs exist in human populations; (ii) *cis*-regulatory variation contributes to both population-selective effects [3], [4] and common disease signals [5], [6], [7]; (iii) a large fraction of species' differentiation is driven by regulatory changes [8], [9], [10].

An extensive number of studies have characterized the level and patterns of regulatory variation and eQTLs over the last decade. eQTLs have been studied in lymphoblastoid cell lines (LCLs) from the HapMap populations [11], [12], [13], [14], [15], in single tissues such as fat, osteoblasts and brain (cortex) [16], [17], [18], as well as across multiple tissues and cell types of the same individual to enable direct comparison of differential *cis*-regulatory effects [19], [20]. Collectively, these studies have demonstrated an abundance of eQTLs in all tissues, and have revealed a substantial tissue- and cell-type specific component of eQTLs. While these studies have partly addressed the degree of population differentiation among human populations for eQTLs [15], [21], they have been limited to only several well-defined populations, and in particular have not contrasted geographically proximate populations. In this study we describe the first analysis of eQTL differentiation among eight human population samples, including three populations from Africa and four admixed populations, using genome-wide expression data for 726 individuals and dense genotyping of over 1.2 million common SNPs; this comprises the most comprehensive dataset and analysis used for this purpose to date.

## Methods

### RNA preparation

Total RNA was extracted from lymphoblastoid cell lines of the 726 individuals of 8 HapMap populations. The numbers of individuals of each population includes: CEU: 109 Caucasians living in Utah USA, of northern and western European ancestry, CHB: 80 Han Chinese from Beijing, China, GIH: 82 Gujarati Indians in Houston, TX, USA, JPT: 82 Japanese in Tokyo, Japan, LWK: 82 Luhya in Webuye, Kenya, MEX: 45 Mexican ancestry in Los Angeles, CA, USA, MKK: 138 Maasai in Kinyawa, Kenya, and YRI: 108 Yoruba in Ibadan, Nigeria (International HapMap Constortium 2005; Coriell, Camden, New Jersey, United States). Two *in vitro* transcription (IVT) reactions were performed as one-quarter scale Message Amp II reactions (Ambion, Austin, Texas, United States) for each RNA extraction using 200 ng of total RNA as previously described [13]. 1.5 µg of the cRNA was hybridized to an array [14]. For RNA extractions, IVT reactions, and array hybridizations, samples were processed in an order randomized with respect to population of origin.

### Gene expression quantification

To assay transcript levels in the cell lines, we used Illumina's commercial whole genome expression array, Sentrix Human-6 Expression BeadChip version 2, [Illumina, San Diego, California, United States, 22]. These arrays utilize a bead pool with ∼48,000 unique bead types (one for each of 47,294 transcripts, plus controls), each with several hundred thousand gene-specific 50mer probes attached.

On a single BeadChip, six arrays were run in parallel as described in [14]. Each of the two IVT reactions from the 726 samples was hybridized to one array each, so that each cell line had two replicate hybridizations. cRNA was hybridized to arrays, and subsequently labelled with Cy3-streptavidin [Amersham Biosciences, Little Chalfont, United Kingdom, as described in 14] and scanned with a Bead Station (Illumina) as previously described in Stranger *et al.* (2005). Samples were processed in an order randomized with respect to population of origin and IVT batch.

### Raw expression data normalization

With the Illumina bead technology, a single hybridization of RNA from one cell line to an array produces on average approximately 30 intensity values for each of 47,294 bead types. These background-corrected values for a single bead type are subsequently summarized by Illumina software and output to the user as a set of 47,294 intensity values for each individual hybridization. In our experiment, each cell line was hybridized to two arrays, thus resulting in two reported intensity values (as averages of the values from the 30 beads per probe) for each of the 47,294 bead types. Hybridization intensity values were normalized on a log_2_ scale using a quantile normalization method [23] across replicates of a single individual followed by a median normalization method across all individuals of the eight populations. These normalized expression data for CEU, CHB, JPT, and YRI were used as input for the expression analysis, while the expression data from the populations with admixture (GIH, LWK, MEX, and MKK) were subjected to an additional correction for this genetic structure (See below).

### Population stratification correction of expression data

The expression data for GIH, LWK, MEX and MKK populations were normalized for admixture using a customized version of EIGENSTRAT which generates principal components on the basis of genetic data [24]. Expression values were adjusted for each population using ten primary axes of variation from that population's corresponding intra-population PCA of the set of whole genome SNP genotypes. This correction for admixture also corrects for relatedness among some of the individuals in a few of the population samples as has been described in [25]. These residual normalized expression values were used as input for the association analysis.

### Correction for known and unknown factors: “REDUCED” dataset generation

We employed a latent variable analysis separately for each population to correct the expression data for known and unknown factors that may influence gene expression in this dataset, with the aim to characterize and compare the properties of these results to those obtained without the correction. This dimension reduction differs from the PCA for admixture (Population stratification correction of expression data) in that it is possible to account for effects of unknown covariates, such as complex batch effects or subtle environmental influences, which can then be factored out of the expression data. The reduced expression data sets were learned using the probabilistic estimation of expression residuals (PEER) framework [26], [27]. In this framework, contributions from known and hidden global factors on gene expression levels are estimated and subtracted out to produce a residual gene expression profile. Parameter estimation is performed using variational learning, an approximate inference algorithm that generalizes expectation maximization.

We used the PEER Bayesian regression and factor analysis modules for each of the 8 populations separately to learn the global effects of known and hidden factors on gene expression. Population and gender indicators were modeled as known global factors, essentially using Bayesian regression. Jointly with modeling these known factors, 32 hidden factors were estimated using Bayesian factor analysis. The prior on the weight precisions that acts as a regularization parameter was set to (21800, 0.022) for both models. These regularization parameter influences the effective number of factors retained after training. Specific settings are the standard ARD from [26], scaled with the total number of probes in the model (see [26] for detailed discussion). All remaining priors were set to uninformative values. The residual values were used as input for subsequent analysis and are referred to throughout the manuscript as ‘REDUCED’ data.

### Selection of probes to analyze

Of the 47,294 probes for which we collected expression data, we selected a set of 21,800 probes for analysis. We included in our analyses each probe that mapped to an Ensembl gene, but not to more than one Ensembl gene (Ensembl 49 NCBI Build 36), and we excluded probes mapping to the X or Y chromosome. The final set of 21,800 probes considered for association mapping corresponds to 18,226 unique autosomal Ensembl genes. We mapped known 1000 genomes common SNPs (MAF>5%) from CEU, CHB, JPT, YRI (August 2010) to all probes. We found that of the 21,800 probes we used in our analysis, 1401 (6.4% of tested probes) overlapped a known common SNP. There is the risk of a SNP-in-probe effect for these overlapping variants, inducing false positive eQTLs. We decided to not simply exclude these probes in an overly-conservative manner, but instead tested for possible enrichment among statistically significant eQTLs and compared the degree of replication across populations for those *cis*-eQTLs with probes to those *cis*-eQTLs without probes.

### Genetic variation

Single nucleotide polymorphisms (SNPs) for the same 726 HapMap version 3 individuals of CEU, CHB, GIH, JPT, LWK, MEX, MKK, and YRI, were selected (Release version 2) for use in the association analyses. Any SNP with MAF>5% in a population and with less than 20% missing data was included. This corresponds to between 1.1 million and 1.3 million SNPs per population.

### Structure of gene expression variation among populations

To quantify population differentiation with respect to gene expression levels, we calculated the statistic V_ST_ for each of the 21,800 probes for each pairwise combination of populations. V_ST_ is a measure of the proportion of expression level variance explained by between-population divergence, and is analogous to the population genetics parameter F_ST_, but for a quantitative trait [28]. For a single probe measured in two populations, V_ST_ is calculated as: (V_T_−V_S_)/V_T_, where V_T_ is equal to the total variance across all individuals of the pair of populations and V_S_ is the average within-population variance weighted by each population sample size. V_S_ = (V_1_*n_1_+V_2_*n_2_)/(n_1_+n_2_), where V_1_ is the within-population variance of population 1, V_2_ is the within population variance of population 2, and n_1_ and n_2_ are the numbers of individuals sampled from population 1 and 2, respectively. V_ST_ values range from 0 to 1, with values near 1 signifying that the majority of gene expression variance for a probe segregates between populations rather than within populations.

To address the question of whether genes of specific functional classes tend to be among those exhibiting highest expression differentiation between populations (any pair of populations), we used the V_ST_ statistic as a determinant of expression differentiation and selected the top 5% of the probes which were significantly differentiated between any two populations. Using this cutoff, we computed a one-sided Fisher's exact probability to determine enrichment of Gene Ontology (GO) terms. The GO p-values were for all pairwise comparisons were combined using Fishers combined probability, to identify those functional categories that across all pairs of populations are consistently among the most diverged between pairs of populations. To address the question of whether genes of specific functions exhibit significant population-specific expression differentiation, we used the V_ST_ score and selected the top 5% of the probes which were significantly differentiated between any two populations. Using this cutoff, we computed a one-sided Fisher's exact probability to determine enrichment of GO terms in the top 5% of probes. For a single population, such as CEU, all GO term p-values from pairwise population comparisons with CEU were combined using the Fisher combined probability and compared to the combination of GO term p-values for the other populations (in this case, excluding CEU). We then filtered from the primary population those GO enrichment terms which were also found significantly differentiated among other populations. From this filtered list, we then selected the top ten GO terms for each population. These top ten values were used as indicative of functions which are significantly differentiated between the primary population and other populations and not between any other populations.

We addressed the question of whether those functions which are significantly differentiated in one population inform differentiation in a closely-related population (i.e., differentiation of function may be shared by closely-related populations). For example, differentiation might be similar for each of CHB and JPT when individually compared to all the other six populations (or for LWK and MKK and YRI). To address this, we assessed the enrichment in the p-value distribution for GO terms predicted in one population to be significant in another population (for each comparison both populations being compared are excluded). For example, we identify the GO terms that are significantly differentiated between CHB and all other populations (excluding JPT) and compare their p-values to the p-value distribution for terms with JPT and all other populations (excluding CHB).

### Association analyses

The eQTL association analysis employed: 1) Normalized log_2_ quantitative gene expression measurements for the 21,800 probes (18,226 unique autosomal genes) from 726 unrelated individuals of each HapMap population assayed on the Illumina Sentrix Human-6 Expression BeadChip, 2) SNP genotypes for the unrelated individuals of each HapMap population with minor allele frequency above 5%.

### Association and multiple-test correction (individual populations)

For each of the selected probes interrogating expression and for each SNP, we fit a Spearman Rank Correlation (SRC) model as previously described [14], [15], [19], [29]. The model was applied to each population separately, and to each of the normalized datasets: 1) the normalized and stratification-corrected expression data, and 2) the ‘REDUCED’ expression data. To assess significance of associations of expression variation to SNP genotype, we performed 10,000 permutations of each expression phenotype (probe) relative to the genotypes. We performed a *cis*-eQTL analysis as follows: We limited the analysis to those probes and SNPs (MAF>5%) where the distance from the genomic location of the transcription start site (TSS) to SNP genomic location was less than or equal to 1 Mb. An association to a gene expression phenotype was considered significant if the p-value from the analysis of the observed data (nominal p-value) was lower than the threshold of the 0.01 tail of the distribution of the minimal p-values (among all comparisons for a given gene) from 10,000 permutations of the expression phenotypes [30]. We calculated the false discovery rate for associations in each population at each threshold on the basis of the number of genes tested and the significance threshold of the permutations. We also estimated FDR by evaluating the degree to which associations that are discovered in one population replicate in another population. For each population, we considered all significant associations (0.01 permutation threshold) and determined whether the SNP-probe pair corresponding to the most significant SNP/Ensembl gene replicated (0.005 nominal and same direction) in at least one other population. We estimated FDR as 1−(the number of genes with replication/total number of significant genes).

### Stepwise association model

To determine whether independent *cis*- regulatory signals exist for a given gene, we applied a stepwise association model as follows: For each probe that had a significant *cis*-eQTL at the 0.01 significance threshold, we regressed out of the expression levels the effect of the most-significant SNP, re-ran the SRC analysis on the rest of the significant *cis*-eQTL SNPs using the resulting expression residuals, and stored those SNPs with p-values more significant than the gene's permutation threshold. This was repeated separately for each probe until there were no SNPs from the initial significant eQTL list left to test (i.e. until none pass the permutation threshold after removing the effect of the most significant SNP at that step). At each iteration step, the most-significant SNP passing the permutation threshold is stored as an independent eQTL. We compared results obtained using the permutation thresholds based on the PCA corrected expression data to those obtained using permutation thresholds based on the residuals determined at each step of the stepwise model. We observed that there was no difference in the number of detected effects (not shown), so we used the thresholds based on the PCA residuals to evaluate p-values across steps.

## Results

### Structure of gene expression variation among populations

We assessed the global landscape of expression using principal components analysis (PCA) (Figure S1). Unlike SNP-based PCA plots for the same populations, all populations in the expression-based PCA plot do not separate distinctly by their continental ancestry. We assessed correlation of principal component (PC) 1 from the SNP-based PCA which separates African/non-African populations against all principal components from the expression PCA and found decay of correlation from the first 50 principal components maximized at PC3 and PC7 highlighting that gene expression differences, while not distinguishable by heredity alone, are partly shaped by it (Figure S2).

To quantify population differentiation with respect to distinct gene expression levels, we calculated the statistic V_ST_ for each of 21,800 probes, corresponding to 18,226 unique autosomal Ensembl genes (see Methods), for each pairwise combination of populations. For each pairwise comparison of populations, the distribution of V_ST_ values was heavily skewed toward values near 0, with a long narrow tail comprised of values between 0 and 1 (Table S1 and Figure S3). Individual pairwise combinations of populations differ with respect to numbers of genes exhibiting high V_ST_ genes (Table S1) such that the amount of V_ST_ between a pair of populations is correlated with the degree of genetic distance; For example, the CHB-JPT combination only has 13 genes with V_ST_ greater than 0.2, whereas the CHB-MKK combination has 4031 genes with V_ST_ greater than 0.2. Together these analyses indicate that the vast majority of genes do not exhibit highly differentiated expression variation between populations, however every pairwise combination of populations has genes with highly structured expression variation. Analysis of the union of probes exhibiting top 5% V_ST_ scores from each pairwise population comparison indicates a significant enrichment of Gene Ontology (GO) terms, including nucleus, protein binding, RNA binding, nucleotide binding, RNA splicing (Table S2). Each of the eight populations also exhibited significant population-specific GO term enrichment when top population-specific V_ST_ scores were analyzed (Table S3). For example, CEU exhibits an enrichment in immune response (GO:0006955, p-value 6.7×10^−6^) and regulation of immune response (GO:0050776, p-value 4.05×10^−5^), indicating strong structure of expression variation for genes of these categories in CEU versus other populations, and that this structure is not seen between any other populations. We also addressed the question of whether those functions which are significantly differentiated in one population inform differentiation in a closely-related population (i.e., differentiation of function may be shared by closely-related populations). For example, differentiation might be similar for each of CHB and JPT when individually compared to all the other six populations (or for LWK, MKK, and YRI). In general, GO terms corresponding to genes which are significantly diverged in expression in one population relative to the others are also diverged in expression in the other, closely-related populations (Figure S4). A caveat of these analyses is that the cell lines of individual populations were initially transformed at different time points and have been subject to differing numbers of passages, so it is possible that some of the population-specific signals reflect technical issues as opposed to true population-level divergence of function. However, when we consider the number of genes with V_ST_ greater than 0.2 in relation to median F_ST_, we do not observe that comparisons involving CEU, the oldest cell lines exhibit unusually high V_ST_ (Figure S5).

### *Cis* associations of gene expression with SNPs

For each of the 21,800 probes (18,226 unique autosomal genes) selected for analysis, we performed a *cis*- association test between expression and common SNP genotypes using a Spearman Rank Correlation (SRC) model (See Methods). The model was applied to each population separately: 1) For the normalized and stratification-corrected expression data, and 2) for the REDUCED expression data. The purpose of presenting both sets of results is to compare the properties of the two sets, as opposed to simply choosing one approach over the other. We analyzed in depth those associations significant at the 0.01 permutation threshold. At this level of significance, we expect roughly 182 genes to have at least one significant association by chance, and we detected 657, 774, 698, 795, 773, 472, 947, 799 genes with a significant association in CEU, CHB, GIH, JPT, LWK, MEX, MKK, and YRI, respectively with a false discovery rate (FDR) of 18–39% per population (Table 1). Similar FDR values were obtained when evaluating the degree of replication of an eQTL discovered in one population by replication in another (see Methods) where FDR was estimated to range from 31% to 40%. A lower FDR was observed in non-African populations as expected, given that only a subset of African eQTLs will be represented in non-African samples. In total, at the 0.01 threshold we detected a non-redundant set of 3,130 genes exhibiting a significant *cis* association in at least one population. At higher stringency (permutation threshold 0.001), the per-population FDR ranged from 4%–11%; with a total of 1,132 genes exhibiting a significant *cis* association in at least one population.

**Table 1:** *Cis*- associations detected with Spearman Rank Correlation analysis of normalized and PCA-corrected expression data.

	permutation threshold			
	0.01		0.001	
	significant genes	FDR	significant genes	FDR
**CEU**	657	0.28	313	0.06
**CHB**	774	0.24	378	0.05
**GIH**	698	0.26	300	0.06
**JPT**	795	0.23	386	0.05
**LWK**	773	0.24	311	0.06
**MEX**	472	0.39	165	0.11
**MKK**	947	0.19	411	0.04
**YRI**	799	0.23	328	0.06
**Nonredundant**	3130		1132	
**≥2 populations**	1074		547	
**8 populations**	63		28	

As *cis*-eQTLs associated with probes overlaying SNPs need to be interpreted with caution, we examined our significant associations for potential artifacts. At the 0.01 permutation threshold, of the 3,292 probes with significant associations (corresponding to 3,130 genes), 249 probes (245 genes), overlaid a SNP. This means that 7.5% of the probes with a significant *cis*-eQTL overlaid a common SNP (compared to 6.5% among all probes), which did not represent a significant enrichment of these probes among our significant associations. We did, however, consider the effect of these associations on across-population sharing of associations, and report levels of sharing both with and without these probes. At the 0.01 permutation threshold, of the 3,130 genes exhibiting a significant *cis* association, 1,074 genes had a significant association in at least two populations, and 63 in all eight populations. This indicates that 34% of genes with a significant *cis*-association had an association in at least two of the populations, and 2% of genes in all eight populations. If we conservatively exclude those probes known to overlap common SNPs, we reduce the number of non-redundant genes exhibiting a *cis*-association to 2,900, but observe that 957 (33%) of the remaining genes had a significant *cis*-association in at least two of the populations, and 54 (2%) in all eight populations. Thus, probes with underlying SNPs are not contributing significantly to our estimates of across-population sharing (i.e., replication) of eQTLs. At higher stringency (permutation threshold 0.001), we note that none of the significant associations involve probes with underlying SNPs, and 48% of genes with a significant *cis*-association had an association in at least two of the populations, and 2% of genes in all eight populations (Table 1).

To increase the power of our analysis we ran the *cis*- association analysis using the REDUCED data (see Methods) and the same analysis parameters, and at the 0.01 permutation threshold detected 1,966, 1,950, 1,984, 2,131, 1,794, 1,131, 2,562, 2,415, genes with a significant association in CEU, CHB, GIH, JPT, LWK, MEX, MKK, and YRI, respectively with a false discovery rate (FDR) of 7–16% per population (Table 2). In total, there is a non-redundant set of 5,691 genes showing a significant *cis* association in at least one population, 3,240 in at least two populations, and 331 in all eight populations. This indicates that 57% of genes with a significant *cis*-association had an association in at least two of the populations, which is an increase over the 34% replication observed using the normalized and PCA-corrrected data (Table 1). As expected given each population's sample size, all significant detected effects are relatively large; the range of Spearman's rho, the correlation coefficient, is 0.338–0.919 for the normalized and PCA-corrected data, and 0.337–0.933 for the ‘REDUCED data’ (Table S4). There is substantial overlap between genes detected from the normalized and PCA corrected data with that of the REDUCED data (Table S5). Of the genes with significant *cis*- associations in the REDUCED data analysis, 70–77% of the genes are novel, i.e., were not identified as having a significant *cis*- association in the normalized and PCA-corrected data analysis, though the vast majority of these significant p-values were close to significance thresholds in the PCA corrected data analysis (Figure S6). The additional *cis*-eQTLs detected in the REDUCED analysis are likely due to the increased sensitivity of the analysis (see [27]). Of the 22 to 35 percent of the *cis*-eQTLs that did not replicate in the REDUCED analysis, a portion were likely artifactual associations in the first analysis, or else weak effects with low power for replication (as evidenced by the lowest replication in the analysis of the smallest population, the MEX). Together, these results demonstrate that by applying dimension reduction to the expression data, we do not introduce bias, rather we increase power and replication.

**Table 2:** *Cis*- associations detected with Spearman Rank Correlation analysis of “REDUCED” data.

	permutation threshold			
	0.01		0.001	
	significant genes	FDR	significant genes	FDR
**CEU**	1966	0.09	1253	0.015
**CHB**	1950	0.09	1218	0.015
**GIH**	1984	0.09	1219	0.015
**JPT**	2131	0.09	1327	0.014
**LWK**	1794	0.10	962	0.019
**MEX**	1131	0.16	528	0.035
**MKK**	2562	0.07	1528	0.012
**YRI**	2415	0.08	1439	0.013
**Nonredundant**	5691		3231	
**≥2 populations**	3240		2023	
**8 populations**	331		179	

### Multiple effects underlying *cis*-eQTLs

To quantify the degree to which multiple *cis*-associated SNPs comprising a given *cis*-eQTL represent multiple, independent effects, we applied a stepwise regression framework to each probe that had at least two significant *cis*-eQTL SNPs at the 0.01 permutation threshold. We identified a total of 33 genes with multiple eQTLs (0.15% of all 21,800 genes tested), corresponding to 1.1% of genes with significant *cis*-eQTLs, or 1.7% of genes overall that had more than two significant *cis*-eQTL SNPs in at least one population. In CEU, fourteen genes exhibited multiple independent *cis*-effects (corresponding to 1.8% of genes with significant *cis*-eQTLs). In total, 10 genes (1.2%), 1 (0.14%), 7 (0.83%), 1 (0.12%), 0 (0%), 7 (0.71%), and 13 (1.5%) with multiple *cis*-eQTLs were detected for CHB, GIH, JPT, LWK, MEX, MKK and YRI respectively (Table S6). Taken together, at the 0.01 permutation threshold for all eight populations, we observed that ∼0–2% of genes with an expression association possess multiple independent *cis*-eQTLs effects. At most, a single gene had five independently associated SNPs, i.e., expression of *TACO1* (Syn *CCDC44*), translational activator of mitchondrially encoded cytochrome c oxidase subunit I, a gene with a role in Leigh Syndrome, was independently associated with five SNPs in LWK.

### Population sharing of *cis*-eQTLs

As described above, at the 0.01 permutation threshold we detected 3,130 genes that had a significant *cis*- association, 1,074 (34%) of which had a significant *cis*-eQTL in at least two populations. We evaluated whether the extent to which pairs of populations shared significant *cis*- associations was related to their distance as defined by SNPs, with the goal of assessing the degree of sharing of functional variation across populations of varying ancestry. Qualitatively, we observed that more closely-related populations tend to share more *cis*- associated genes than more distantly-related populations (Figure S7). We further considered sharing using parsimony. Assessing eQTLs discovered at the 0.01 permutation threshold and shared at least at the 0.1 permutation threshold, we used an estimate of the general population structure of these eight populations to identify significant subpopulation sharing between CHB+JPT, CEU+GIH and CEU+GIH+JPT+CHB (Figure S8). For eQTLs estimated as ancestral (or recurrent) to all populations using this methodology we did not find any enrichment in particular functional categories (no GO terms had a 0.05 significance after Bonferroni correction).

**Figure 1:**
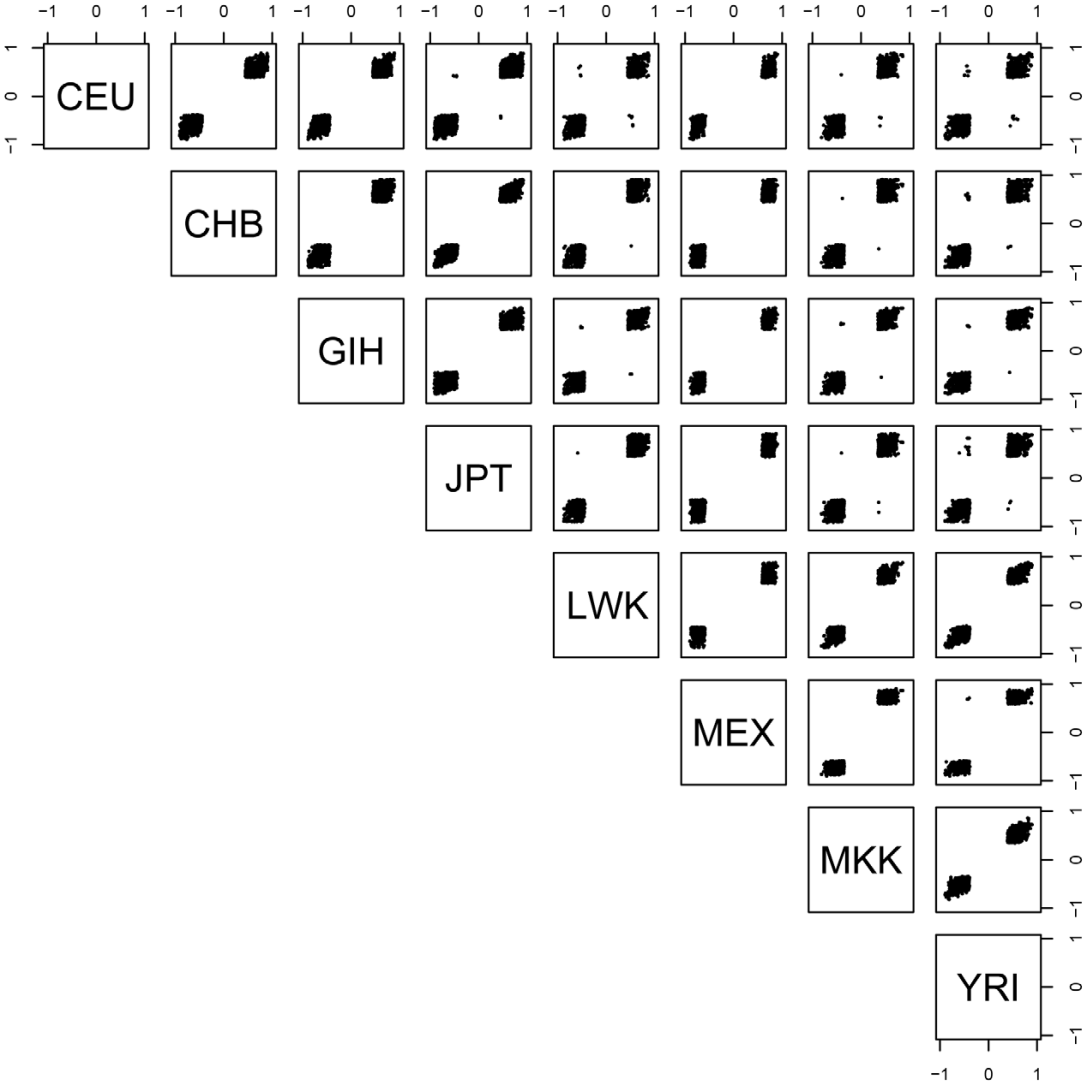
Spearman's rho for each significant SNP-probe *cis*- association shared by at least two populations. Shown are plots of rho for significant associations (permutation threshold 0.01) for each pairwise combination of populations. Within a panel, dots shown in upper left and lower right quadrants indicate significant SNP-probe associations where the allelic direction of the association is in opposite directions in the two populations being compared.

For each pairwise combination of populations, we examined those SNP-probe pairs that were significant in both populations and determined the proportion that had the allelic effect in the same direction in both populations. As described above, at the 0.01 permutation threshold we detected 1,074 genes with a significant *cis*- association in at least two populations. Among the 28 population pairs, we observed 98.9–100% concordance of allelic direction (Figure 1). Evaluation of allelic direction concordance from the ‘REDUCED data’ analysis produced nearly identical results (not shown). These results suggest that regulatory variation affects gene expression in the same direction across populations.

**Figure 2:**
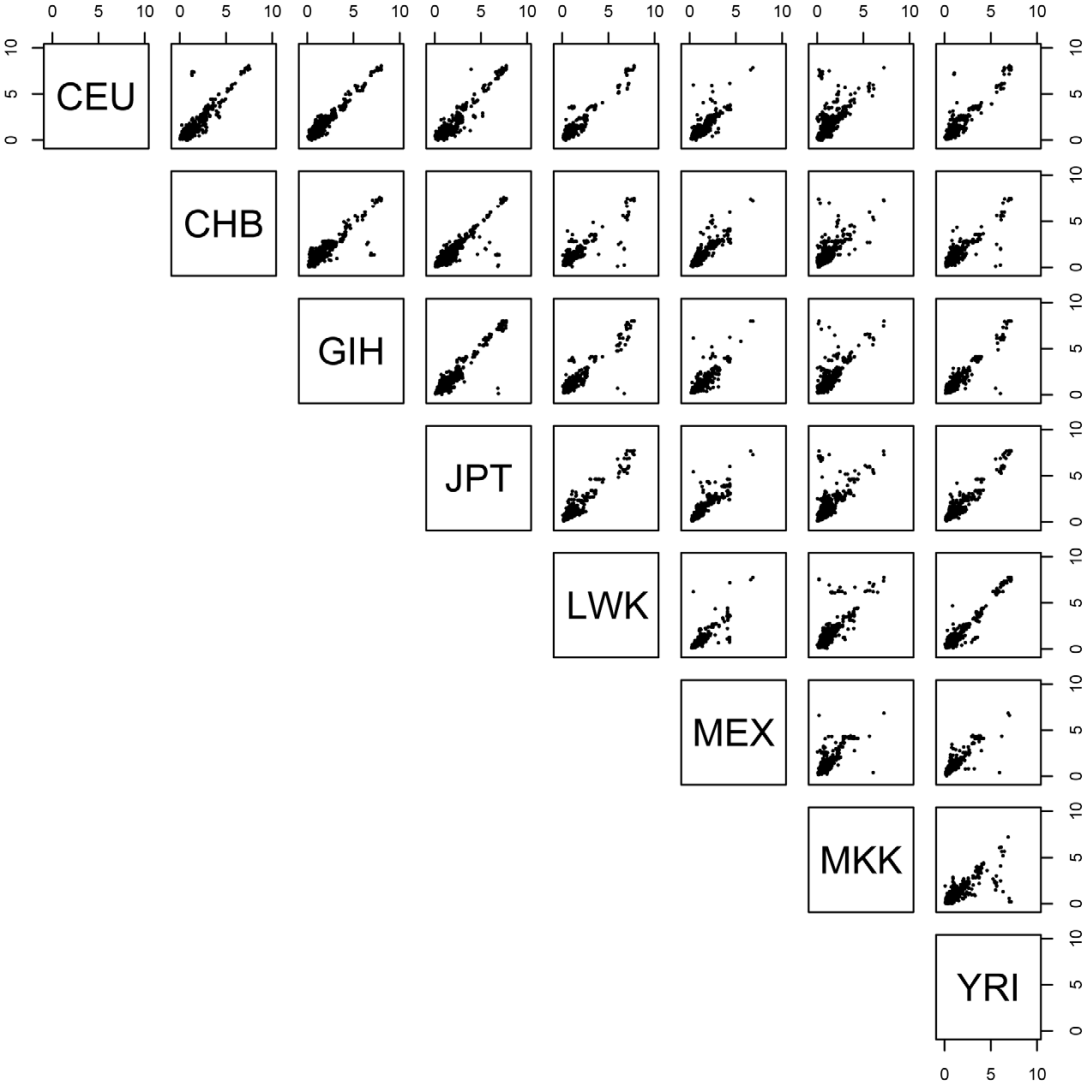
Expression level fold-change for significant SNP-probe *cis*- associations shared by pairs of populations. Shown are plots of the absolute value of expression level fold-change between median expression levels of homozygote classes for significant associations (permutation threshold 0.01) for each pairwise combination of populations. Within a panel, deviating from the 1 to 1 line (lower left to upper right) indicates differences in expression level fold-change (effect size) on log_2_ scale in the two populations being compared.

To quantify the degree of concordance of effect size across populations, for those SNP-probe associations significant in multiple populations, we asked whether the SNP exerts the same effect size in each of the populations, as quantified by expression level fold-change differences between homozygote genotype categories in each of the populations. We observe that the effect size (fold difference between homozygotes of the two different genotypic states of a SNP) is shared between any two populations when the association is also shared (Figure 2), and furthermore, larger effect sizes were slightly more likely to be shared (Figure S9). In addition, for SNP-probe pairs discovered in one population, if we consider the p-value distribution in the other seven for the same SNP-probe pairs, we observe extensive enrichment of low p-values as indicated by the fraction of expected true positives pi1 (Figure S10), indicating that our threshold-based estimates of across-population *cis*-eQTL sharing are underestimates. This result, paired with the result that effect sizes (fold-change) are similar among populations, suggests that the driving force behind the discovery of an eQTL in one population but not another is mainly due to allele frequency differences and not due to differences in absolute effect size.

### Genomic properties of eQTLS

**Figure 3:**
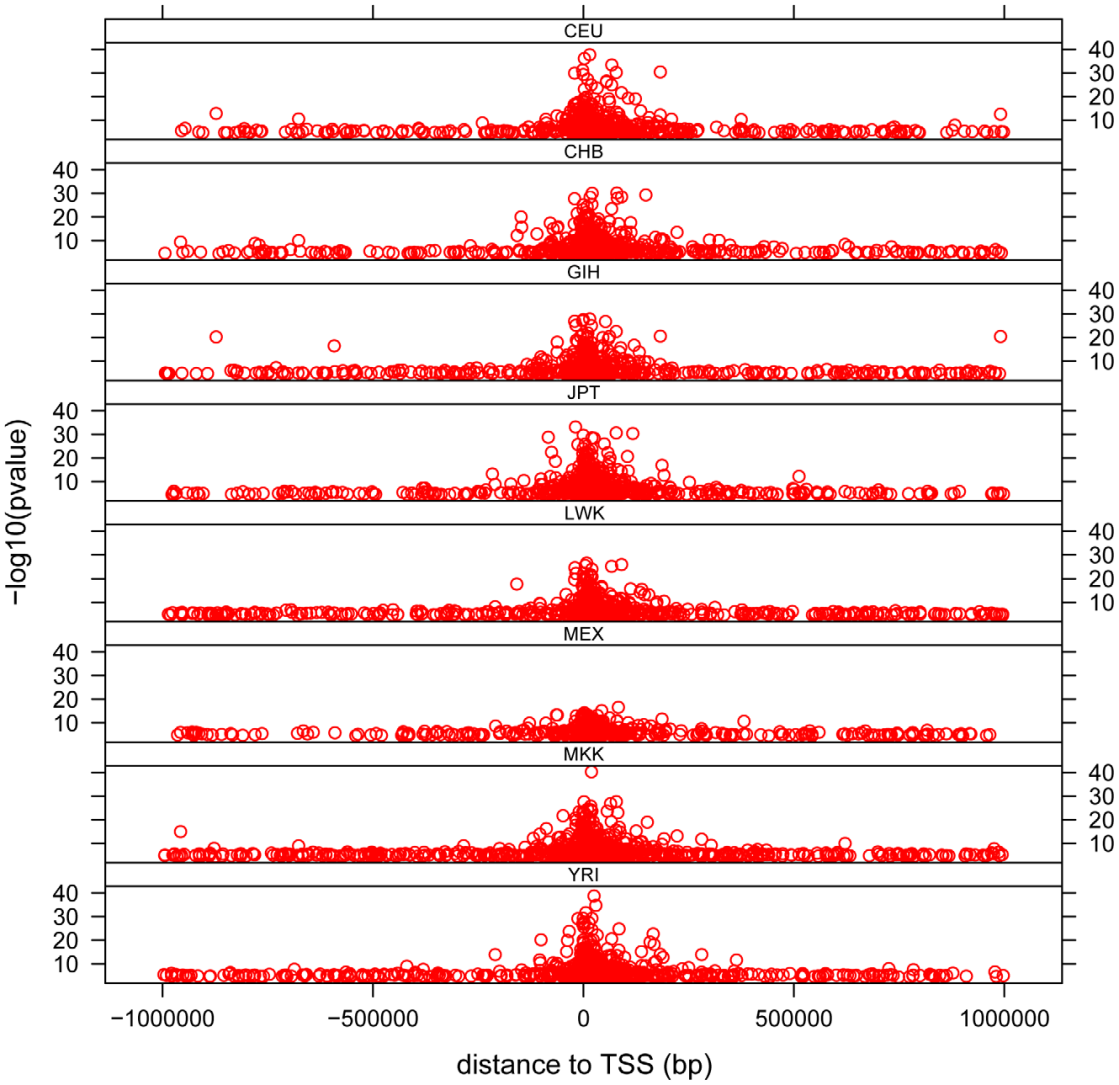
Distribution of *cis*- associations in each population relative to the transcription start site (TSS). −log_10_ of the p-value is plotted against distance measured in base pairs from the associated SNP to the TSS. Each dot represents the most significant SNP for a significant gene (permutation threshold 0.01) in a population. Each panel represents a different population.

**Figure 4:**
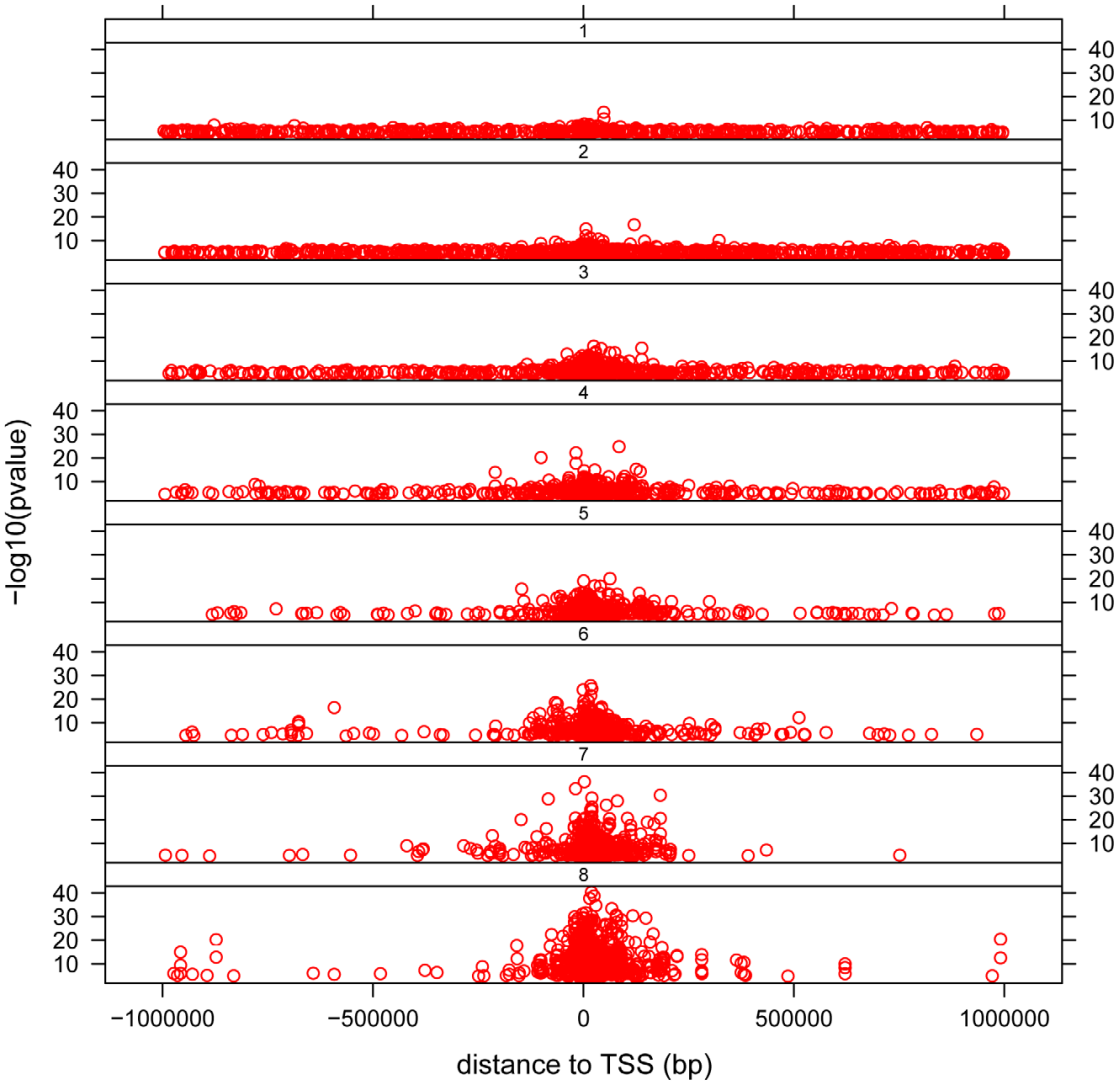
Distribution of *cis*- associations relative to the transcription start site (TSS) and in relation to population sharing. −log_10_ of the p-value is plotted against distance measured in base pairs from the associated SNP to the TSS. Each dot represents the most significant SNP for a significant gene (permutation threshold 0.01) in a population. Panels separate associations that were significant in one population, two populations, etc. All populations are lumped together.

The distribution of *cis*- associations relative to the transcription start site (TSS) shows that the majority of association signals are approximately symmetrically centered on the TSS (Figure 3), with the majority within 100 Kb of the TSS, as has been previously observed [13], [14], [15], [19], [29]. Significant associations extend out to 1 Mb (the limit tested in this analysis), with the strongest statistical signals located directly at the TSS. Note the smallest population sample, MEX, provided the weakest statistical signals as expected. We observe a pattern to this distribution when we partition associations into categories based on the number of populations in which the gene is found to have a significant *cis*- association. We observe that for those genes found to have a significant *cis*-eQTL in only one population, the distribution of most-significantly associated SNPs are uniformly distributed throughout the 2 Mb window (Figure 4 and Figure S11). For genes with significant *cis*-eQTL associations in all eight populations, the distribution is centered on the TSS, with few associations extending beyond +/−200 Kb from the TSS. As population sharing increases from genes with significant associations in only one population to genes with significant associations in all eight populations, we see a gradual tightening of the distribution around the TSS. This is true for single populations (Figure S11) and when examined in aggregate across populations (Figure 4). This is unlikely to be driven simply by false positive associations that fail to replicate, as the tightening pattern is observed even when comparing associations shared in six versus seven versus eight populations, which are themselves unlikely to be false positives. This observation suggests that the genomic distribution of *cis*-regulatory variants with respect to their contribution to genome function is different even when deviations of allele frequency are taken into account.

We also observed that a substantial number of SNPs were associated with more than one gene. A total of 264 genes have eQTLs at the 0.01 permutation threshold organized in clusters of 2 or more genes, where the eQTL is identical among genes, suggesting same functional variant. Of these, 52 clusters of 2 or more genes were observed (and therefore replicated) in at least two populations and the distance to TSS of such eQTL-SNPs was larger relative to all other eQTL-SNPs for single genes. This signal suggests the presence of regulatory domains that influence multiple genes in a coordinated fashion and these tend to do so from long distances. Such coordinated regulation from distance is well known in the HOX cluster [31], but our data suggests that this may be a more general phenomenon.

### eQTLs and disease

The integration of eQTL results with GWAS has been proposed as a way to move toward biological and mechanistic understanding of complex trait etiology [32], [33], and is already achieving success [e.g.,34]. It has also recently been demonstrated that genome-wide association signals are enriched for eQTLs [5], [6], [7]. We compared our *cis*-eQTL results to the National Institute of Health's catalog of genome-wide association studies [35], [36], and found that of 4,772 GWAS SNPs representing 475 traits (available as of August 4, 2011), 62 SNPs were also the most-significant SNP of a *cis*-eQTL in at least one population (0.01 permutation threshold, ‘REDUCED’ analysis). These 62 SNPs associate with expression of 57 Ensembl genes, and 51 traits, including Alcohol dependence, Crohn's disease, Coronary Heart Disease, HDL cholesterol, Prostate Cancer, Trigylcerides, and many others (Table S7). The majority of GWAS studies have been performed in populations of Caucasian ancestry, however the overlap of GWAS SNPs to the strongest-associated *cis*-eQTLs did not reflect this; instead all populations were represented (CEU: N = 14 SNPs, CHB: N = 10, GIH: N = 9, JPT: N = 21, LWK: N = 8, MEX: N = 10, MKK: N = 9, YRI: N = 8). Of the 62 SNPs that were the most significant *cis*-eQTL for a given gene as well as associated to a trait in the GWAS catalog, we observed that 15 (∼24%) were the most significant SNP of the same gene in at least one additional population, a proportion which is likely an underestimate of the true value, given that the same SNPs weren't necessarily tested in all populations. We asked whether across-population replication of the most significant SNP per gene differed depending on whether that SNP was a GWAS SNP, and observed no difference (Fisher's 2-tailed p-value 0.128). The eQTLs identified through our analyses contribute significantly to the available functional regulatory data that could be used to fine-map and elucidate the function of genetic variants contributing to complex phenotypes.

## Discussion

We have performed a comprehensive study of *cis*- regulatory variation in a single cell type of individuals comprising a diverse set of eight human populations. The analysis of the genetics of nearly 20,000 gene expression phenotypes in such a number of human population samples allows us to identify large numbers of functionally variable regulatory regions in the human genome, as well as to estimate the degree of an aspect of functional variation that has not been assessed before. We find that at least 20% of the genes tested in our analysis have a common *cis*-eQTL in at least one population, and we detect extensive sharing of eQTLs across human populations even with fluctuations in allele frequencies, while there is also substantial non-genetic variance in gene expression levels. Overall, our data show that across all populations, there is an enrichment among population differentiated genes for those genes involved in regulatory function, while each population has unique sets of sets of genes, and categories of gene function, whose expression levels differentiate that population.

In accordance with previous eQTL studies, we observe a symmetric distribution of eQTLs around the transcription start site (TSS), with the strongest, most highly replicated, signals directly at the TSS. The simplest explanation for the relationship between distance to TSS and replication across populations is one of statistical power, as the long-distance smaller effects are less likely to be replicated. However we cannot exclude the possibility that these patterns, at least in part, reflect true biology such as the nature of long-distance enhancers. We applied factor analysis to the gene expression data to account for global non-genetic effects on the expression profiles and increased our power to detect eQTLs, especially those of smaller effect. The additional *cis*-eQTL associations detected using the residual gene expression profiles (‘REDUCED’ data) have very similar characteristics to those detected using the straightforward normalized and PCA-corrected data, however the degree of across-population replication was higher for the ‘REDUCED’ data, which provides confidence that the method is not simply adding false positive associations. Indeed, this demonstrates that by applying dimension reduction, we do not introduce bias, rather we increase power and replication. For associations detected in more than one population, we find nearly perfect concordance of allelic direction across populations, and find that the absolute allelic effect size, as estimated by the fold-change between the two homozygote classes, remains the same across populations, suggesting little in the way of modification of eQTL effects across populations. The last two results support the idea that while eQTLs may explain different degrees of population variance depending on their frequency in each population [21], the absolute effects on each individual are the same. Given that many GWAS signals are likely to be eQTLs [5], [6], it is likely that many of the GWAS signals discovered in one population may be transferable at the level of the absolute individual risk to other populations. One final observation is that we were able to refine our signals at individual loci to identify putative independently-acting *cis*-eQTLs.

Our study represents the most genetically diverse eQTL study undertaken in humans to date, and has revealed extensive *cis*-regulation of gene expression in a single cell type. Taken together, our results suggest extensive *cis*- regulatory variation in humans, much of which will be uncovered as additional cell types are analyzed under a variety different cellular and developmental conditions, in larger numbers of individuals representing an even wider representation of human genetic diversity. Already these analyses contribute to the functional annotation of the human genome, and as a resource we have provided a list of variants that are associated with complex traits and are also the most significant SNP for an eQTL in this cell type. Given the genome-wide scale of these data and the diversity of populations surveyed, these eQTLs may assist in fine-mapping causal variants for complex traits and provide testable hypotheses for the mechanism underlying significant GWAS associations. Finally, our results reveal substantial diversity in frequency of regulatory variants among populations, with future work to understand how that spectrum has been shaped by selective and demographic processes, and how these functional variants contribute to higher order phenotypes, including those of health and disease.

### Accession numbers

The expression data reported in this paper have been deposited in the Array Express (http://www.ebi.ac.uk/arrayexpress/) database (Series Accession Number E-MTAB-264). Furthermore, all eQTL results have been stored in the searchable online GENEVAR eQTL database [37].

## Supporting Information

Figure S1 Principal components analysis (PCA) of gene expression data. Gene expression data for each of the 21800 probes was analyzed using PCA. Here we plot PC1 versus PC2-9. Subtle separation due to ancestry is apparent but is not a dominating feature of expression differences suggesting that environmental or experimental effects are having a non-negligible effect in shaping gene expression differences among all individuals.
(TIF)

Figure S2 Identifying the population component of total expression variability. To determine at which point ancestry affects the landscape of gene expression, we plotted the correlation of all obtainable expression principal components against SNP marker-based PCA component 1 (which separates African versus non-African populations). In the plot below, one can see that correlation is enriched for stronger components (the first 50 components) and maximized at components 2 and 7. We further assessed this for only those probes with significant *cis*-eQTLs, and as expected, these probes are enriched sooner for correlation with their expression PCs against marker-based PC1. This supports the conclusion that population differentiation affects individual genes in different ways.
(TIF)

Figure S3 V_ST_ distribution for 21,800 probes compared between a) CEU and CHB (median 0.01; mean 0.04), and b) CEU and MKK (median 0.03; mean 0.04).
(TIF)

Figure S4 The degree to which significantly diverged gene expression in one population is also divergent in another population. We sought to determine the degree to which significantly diverged gene expression in one population is also divergent in another population. To assess this we calculated the p-value enrichment for GO terms which are significantly associated with expression differentiation in one population in another population (for each comparison both populations are excluded). In general, GO terms corresponding to genes which are significantly diverged in expression in a reference population relative to the others are also diversified in expression within the other populations (excluding the reference population). As an example, we consider the GO terms that are significantly enriched in high V_ST_ genes between CEU and 6 other populations (here excluding YRI; p-values are combined for the 6 populations using Fishers combined probability). We then look at the p-value distribution in YRI for GO terms that are significantly enriched in high V_ST_ genes between YRI and the 6 other populations (excluding CEU). This example is shown in bold green in the top left panel. Interestingly, the admixed populations (GIH, MKK, LWK and MEX) appear to not be well correlated with any other population suggesting that the differentiation of gene expression is unique in these populations.
(TIF)

Figure S5 For each pairwise population comparison, the number of genes with V_ST_ greater than two versus median F_ST_ across all SNPs.
(TIF)

Figure S6 −log10 (p-value) for SNP-probe pair representing the most significant SNP per Ensembl gene (significant at 0.01 permutation threshold) in the REDUCED analysis and the corresponding the −log10 (p-value) for the same SNP-probe pair in the analysis of the normalized and PCA-adjusted data. Panels correspond to a) CEU, b) CHB, c) GIH, d) JPT, e) LWK, f) MEX, g) MKK, h) YRI.
(TIF)

Figure S7 Counts of Ensembl genes with *cis*- associations significant in pairs of populations. Analysis of associations significant at the 0.01 permutation threshold are shown above the diagonal. Analysis utilized normalized and PCA-corrected expression data.
(TIF)

Figure S8 *Cis*-eQTL sharing across populations using parsimony. Average F_ST_ estimates were determined every 10000 SNPs across all pairwise combination of populations and used to create a distance matrix which was assembled into a tree using Neighbour Joining and Matrix Representation with Parsimony. eQTLs were assigned to the tree based on their discovery in at least one population at the 0.01 permutation threshold and then at the 0.1 permutation significance in any additional population. Only one association was selected for each gene; this was done at random. We then created 100 random trees based on reassigning at random the populations at the leaves. Compared to these random trees, the extent of eQTL sharing was increased for some nodes within the observed tree (nodes which have more eQTL sharing than observed in 95% of the random trees are highlighted with red text; this sharing is defined at the same node to respect the overall tree topology, which is maintained, and the respective probability of finding sharing given this topology) . Since this permutation strategy may be overly conservative due to the fact that it does not randomize sharing between populations we also investigated using a different randomization strategy where we randomly assigned the shared populations for each association. Here, we observed not only the previously observed pattern but also observed that the extent of eQTL sharing was increased for nodes within the African subtree (these additional nodes are in black bold text; significance was again defined as excess of sharing compared to 95% of the random trees). These results highlight enrichment of eQTL sharing that are consistent with population structure.
(TIF)

Figure S9 Distribution of effect sizes conditioned on population sharing. We stratified fold change between heterozygotes for 0.1 eQTLs by the number of populations that the eQTL was discovered in at the same threshold. We observed that larger effects are more frequently discovered in multiple populations (Pearson correlation CEU 0.04 p = 1.31e-04, CHB 0.21 p = 2.56e-116, GIH 0.12 p = 1.99e-29, JPT 0.16 p = 3.44e-67, LWK 0.06 p = 7.84e-06, MEX 0.18 p = 6.71e-47, MKK 0.17 p = 1.30e-46, YRI 0.10 p = 2.68e-15).
(TIF)

Figure S10 Distribution of corresponding CEU significant (0.01 permutation threshold) *cis*-association p-values in each of the other seven populations. Left panels display the distribution of all corresponding SNP-probe pair p-values that were significant in CEU, and the right panel shows the distribution of SNP-probe pair p-values after removing those associations that were significant in both CEU and the test population. When the analysis was performed with each other population as the reference, the results remain the same.
(TIF)

Figure S11 Distribution of *cis*- associations relative to the transcription start site (TSS) and in relation to population sharing (i.e., whether the gene had a significant association in a single population (1 pop), two populations (2 pops), etc. Shown is −log_10_ of the p-value plotted against distance from SNP to TSS for each population. Each dot represents the most significant SNP for a significant gene (permutation threshold 0.01) in a population
(TIF)

Table S1 Summary of the distribution of VST values per probe for all pairwise population
comparisons, including the number of genes with VST greater than 0.2.
(PDF)

Table S2 The top 20 most differentiated Gene Ontology functions between all populations.
(PDF)

Table S3 For each population, the top 10 most differentiated Gene Ontology functions between
the primary population and other populations (and not between any other populations).
(PDF)

Table S4 Range of Spearman's rho for a) cis- associations detected using ‘REDUCED’ data,
and b) Spearman's rho for cis- associations detected using normalized and PCA-corrected data.
(PDF)

Table S5 Overlap of cis- associated genes that were detected using: 1) normalized and
PCA-corrected gene expression data, and 2) ‘REDUCED’ data.
(PDF)

Table S6 Number of Ensembl genes with independent cis-eQTLs at the 0.01 permutation
threshold, as determined by stepwise association model.
(PDF)

Table S7 Overlap of the most significant cis -eQTL SNP per gene per population (‘REDUCED’ permutation threshold 0.01) with GWAS SNPs from NHGRI GWAS Catalog (Accessed 8/4/2011).
(PDF)
