# Supplementary figures and images for: Patterns of Cis Regulatory Variation in Diverse Human Populations
Source: PLoS Genet. 2012 Apr 19;8(4):e1002639. doi: 10.1371/journal.pgen.1002639 (PMC3330104; doi:10.1371/journal.pgen.1002639)

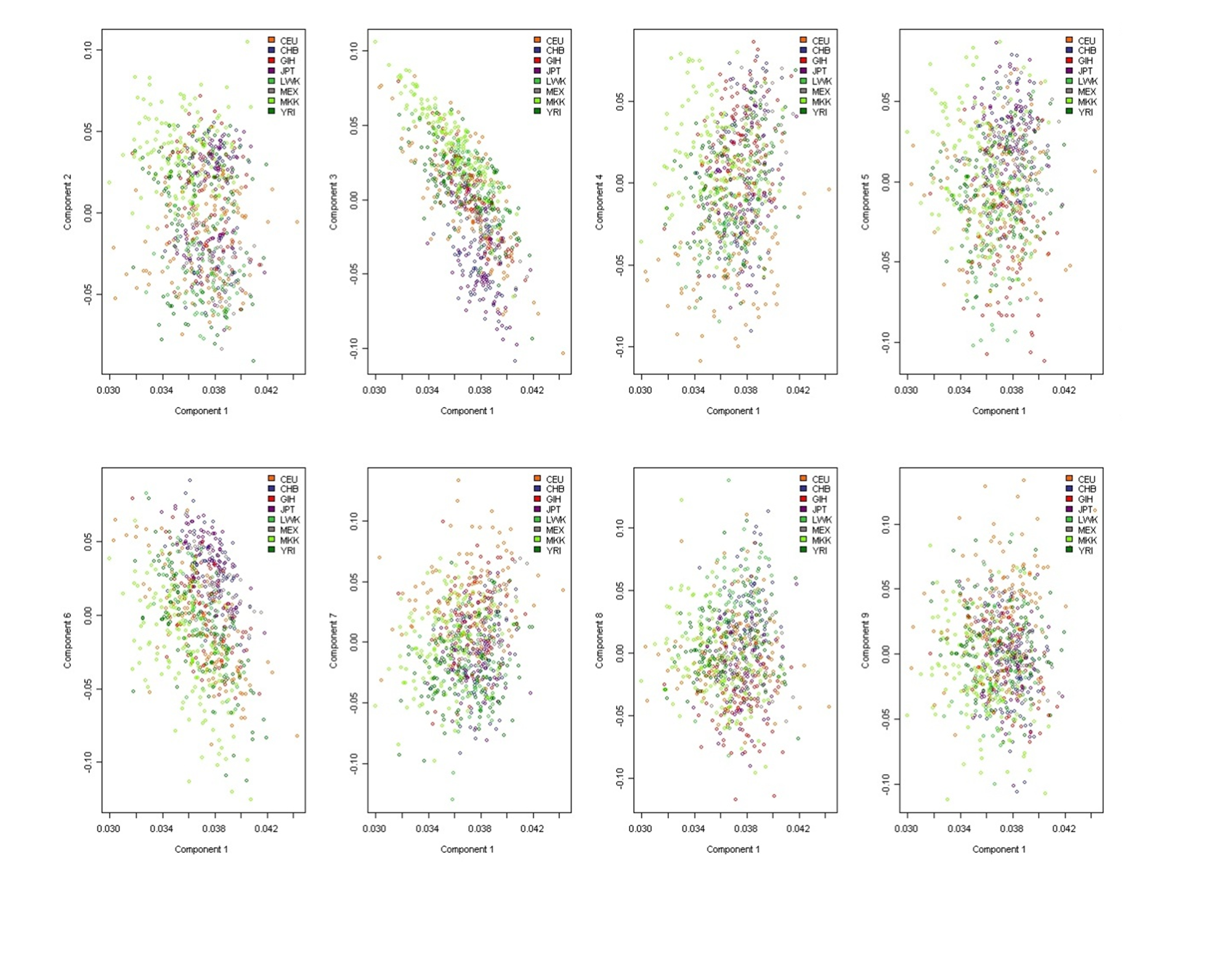

Supplement: Figure S1 — Principal components analysis (PCA) of gene expression data. Gene expression data for each of the 21800 probes was analyzed using PCA. Here we plot PC1 versus PC2-9. Subtle separation due to ancestry is apparent but is not a dominating feature of expression differences suggesting that environmental or experimental effects are having a non-negligible effect in shaping gene expression differences among all individuals. (TIF) [file pgen.1002639.s001.tif]

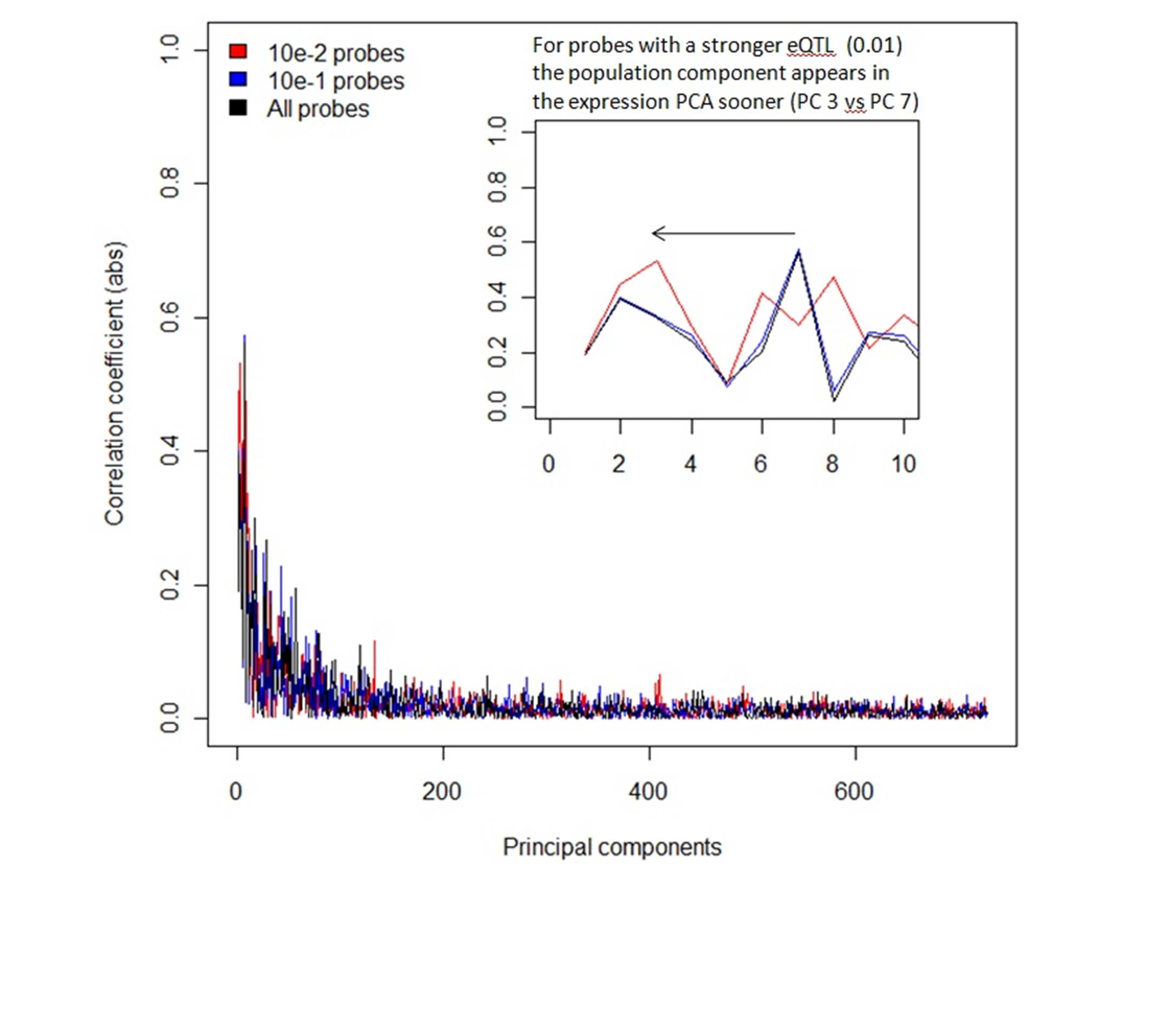

Supplement: Figure S2 — Identifying the population component of total expression variability. To determine at which point ancestry affects the landscape of gene expression, we plotted the correlation of all obtainable expression principal components against SNP marker-based PCA component 1 (which separates African versus non-African populations). In the plot below, one can see that correlation is enriched for stronger components (the first 50 components) and maximized at components 2 and 7. We further assessed this for only those probes with significant cis-eQTLs, and as expected, these probes are enriched sooner for correlation with their expression PCs against marker-based PC1. This supports the conclusion that population differentiation affects individual genes in different ways. (TIF) [file pgen.1002639.s002.tif]

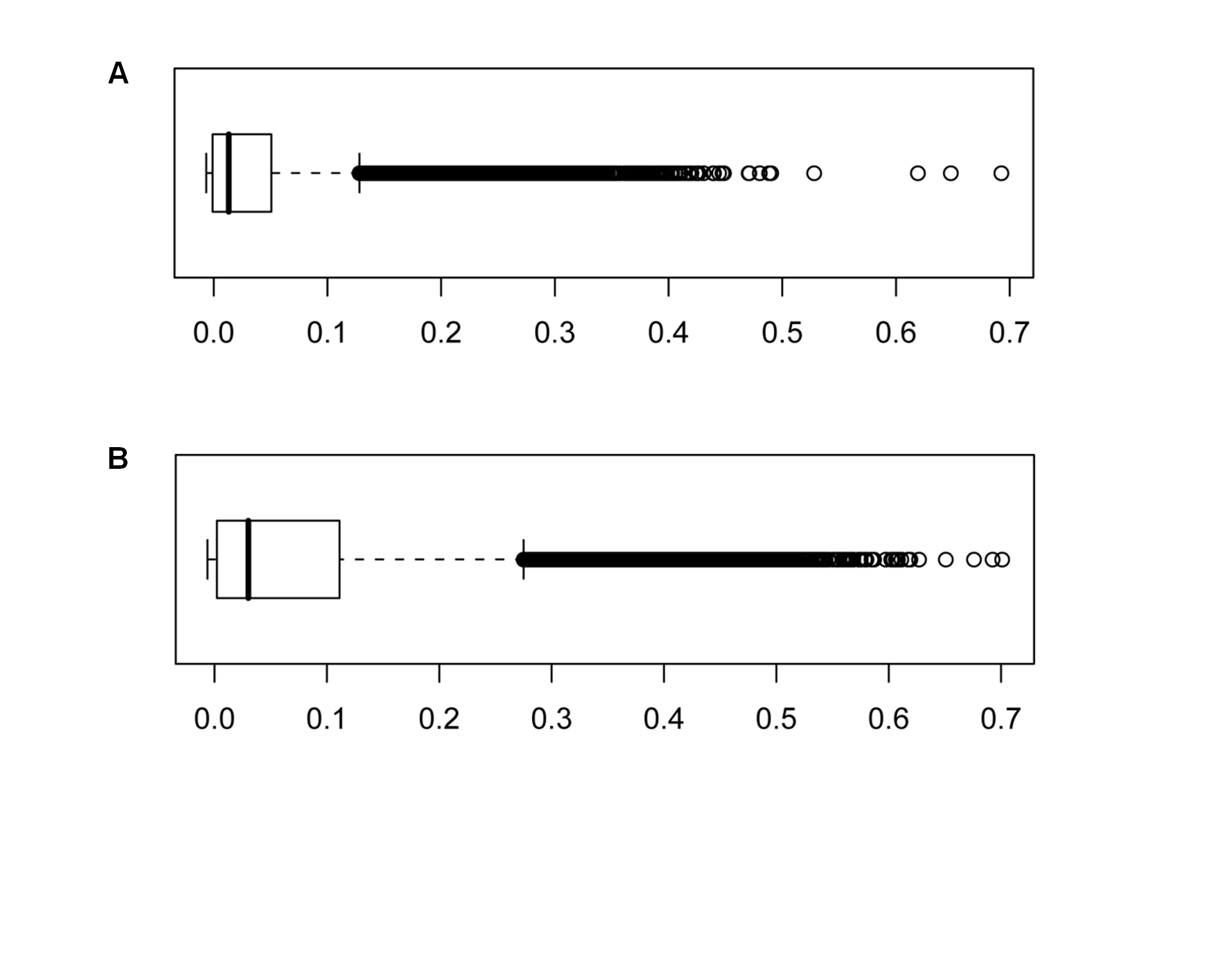

Supplement: Figure S3 — VST distribution for 21,800 probes compared between a) CEU and CHB (median 0.01; mean 0.04), and b) CEU and MKK (median 0.03; mean 0.04). (TIF) [file pgen.1002639.s003.tif]

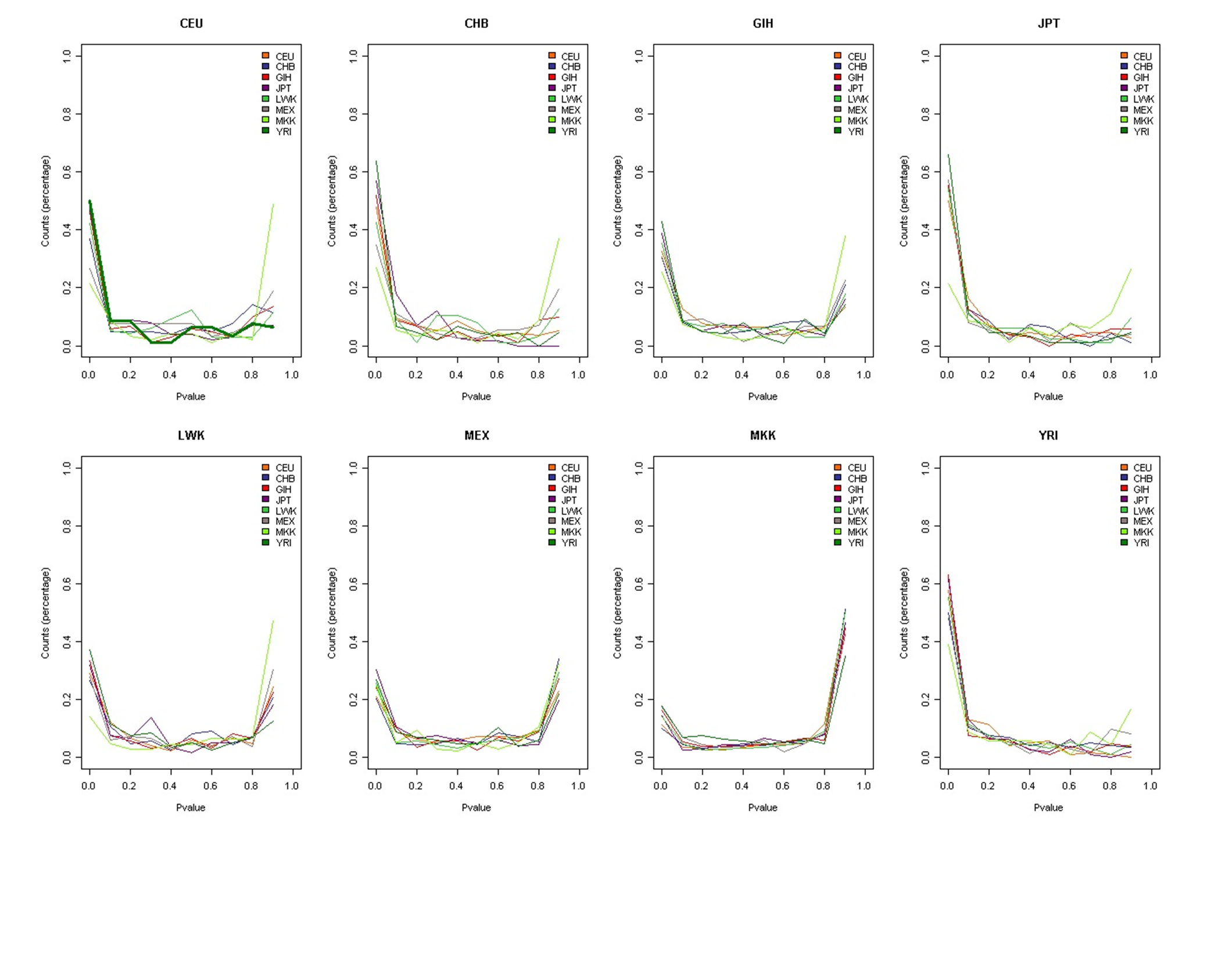

Supplement: Figure S4 — The degree to which significantly diverged gene expression in one population is also divergent in another population. We sought to determine the degree to which significantly diverged gene expression in one population is also divergent in another population. To assess this we calculated the p-value enrichment for GO terms which are significantly associated with expression differentiation in one population in another population (for each comparison both populations are excluded). In general, GO terms corresponding to genes which are significantly diverged in expression in a reference population relative to the others are also diversified in expression within the other populations (excluding the reference population). As an example, we consider the GO terms that are significantly enriched in high VST genes between CEU and 6 other populations (here excluding YRI; p-values are combined for the 6 populations using Fishers combined probability). We then look at the p-value distribution in YRI for GO terms that are significantly enriched in high VST genes between YRI and the 6 other populations (excluding CEU). This example is shown in bold green in the top left panel. Interestingly, the admixed populations (GIH, MKK, LWK and MEX) appear to not be well correlated with any other population suggesting that the differentiation of gene expression is unique in these populations. (TIF) [file pgen.1002639.s004.tif]

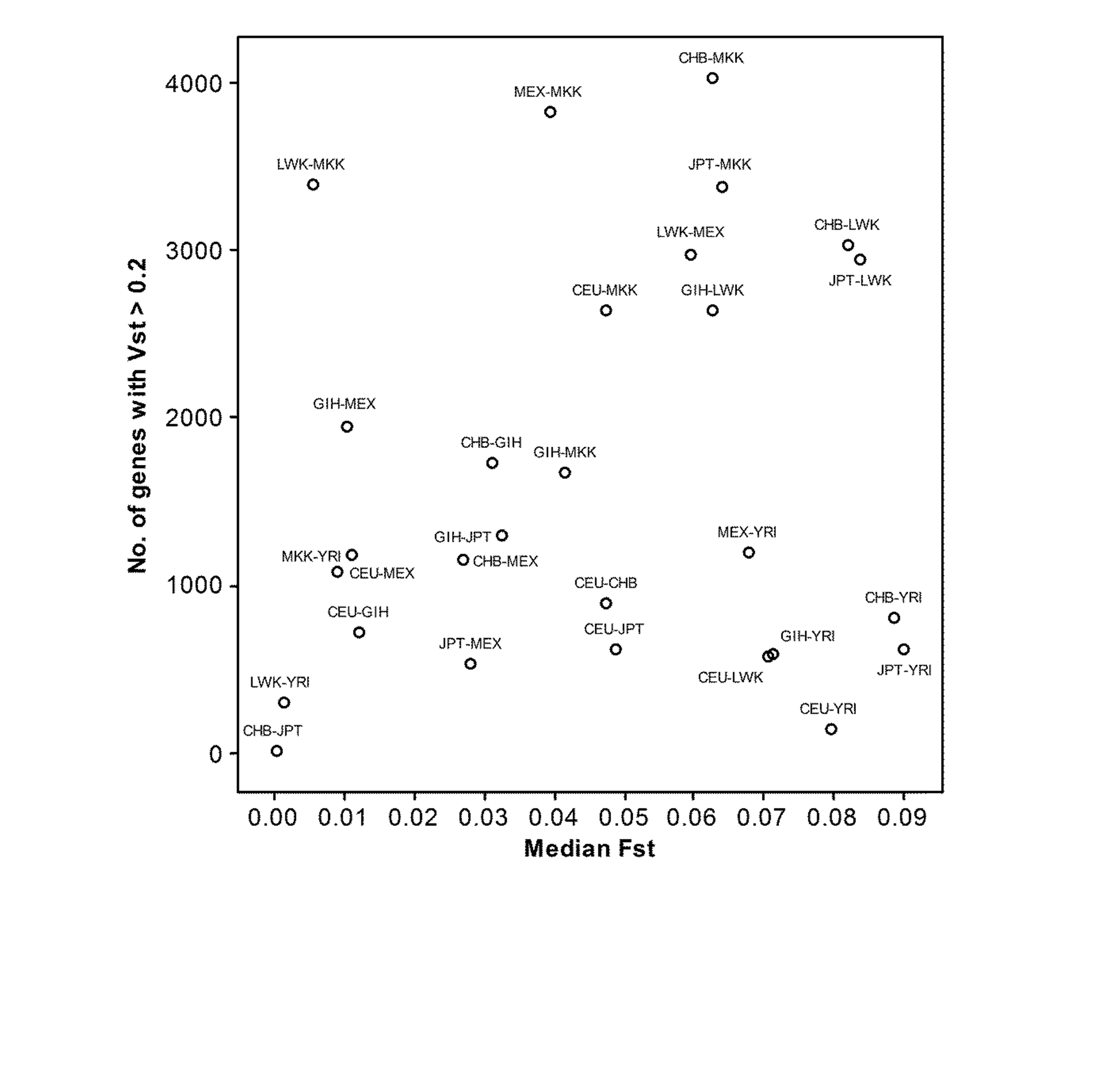

Supplement: Figure S5 — For each pairwise population comparison, the number of genes with VST greater than two versus median FST across all SNPs. (TIF) [file pgen.1002639.s005.tif]

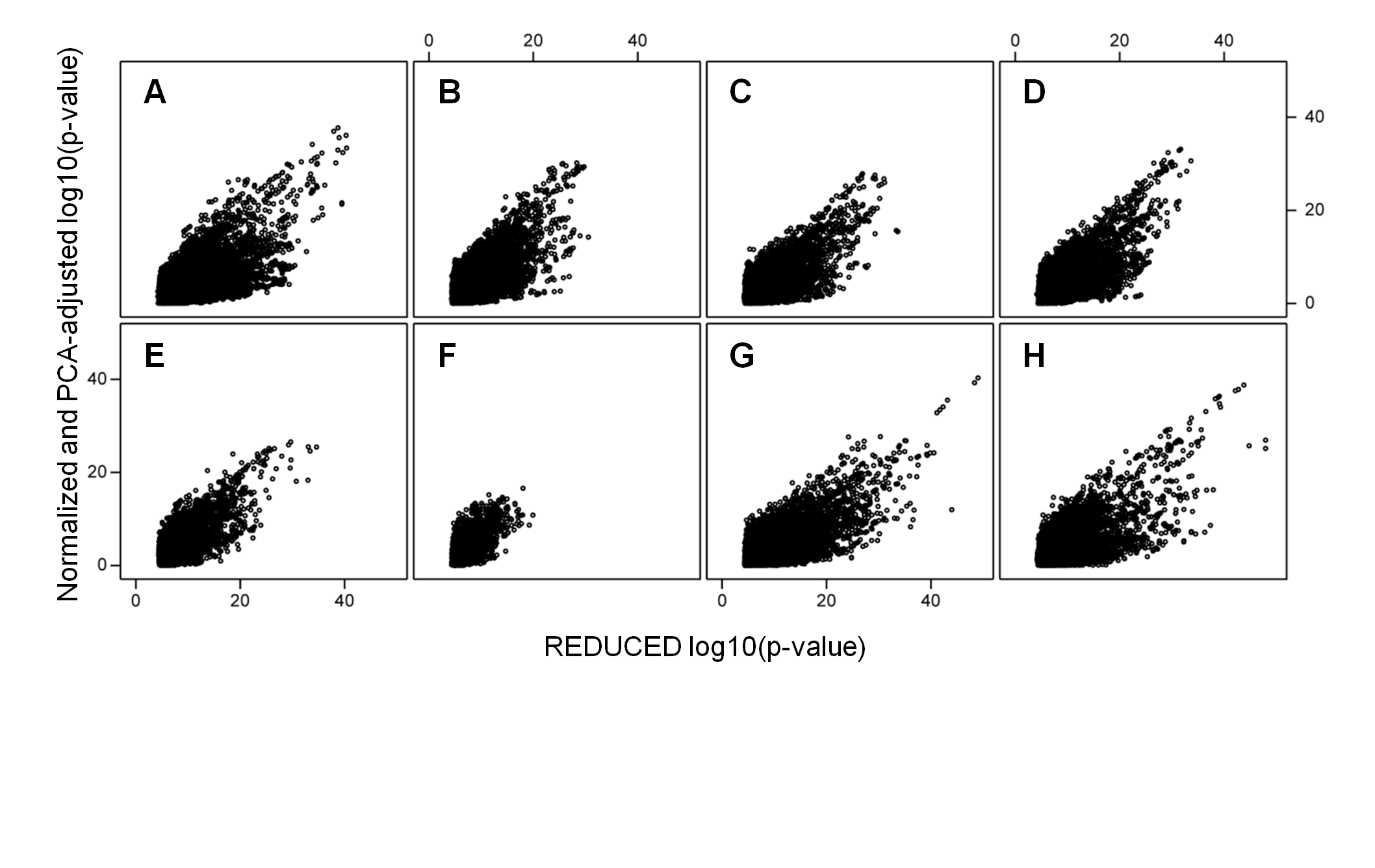

Supplement: Figure S6 — −log10 (p-value) for SNP-probe pair representing the most significant SNP per Ensembl gene (significant at 0.01 permutation threshold) in the REDUCED analysis and the corresponding the −log10 (p-value) for the same SNP-probe pair in the analysis of the normalized and PCA-adjusted data. Panels correspond to a) CEU, b) CHB, c) GIH, d) JPT, e) LWK, f) MEX, g) MKK, h) YRI. (TIF) [file pgen.1002639.s006.tif]

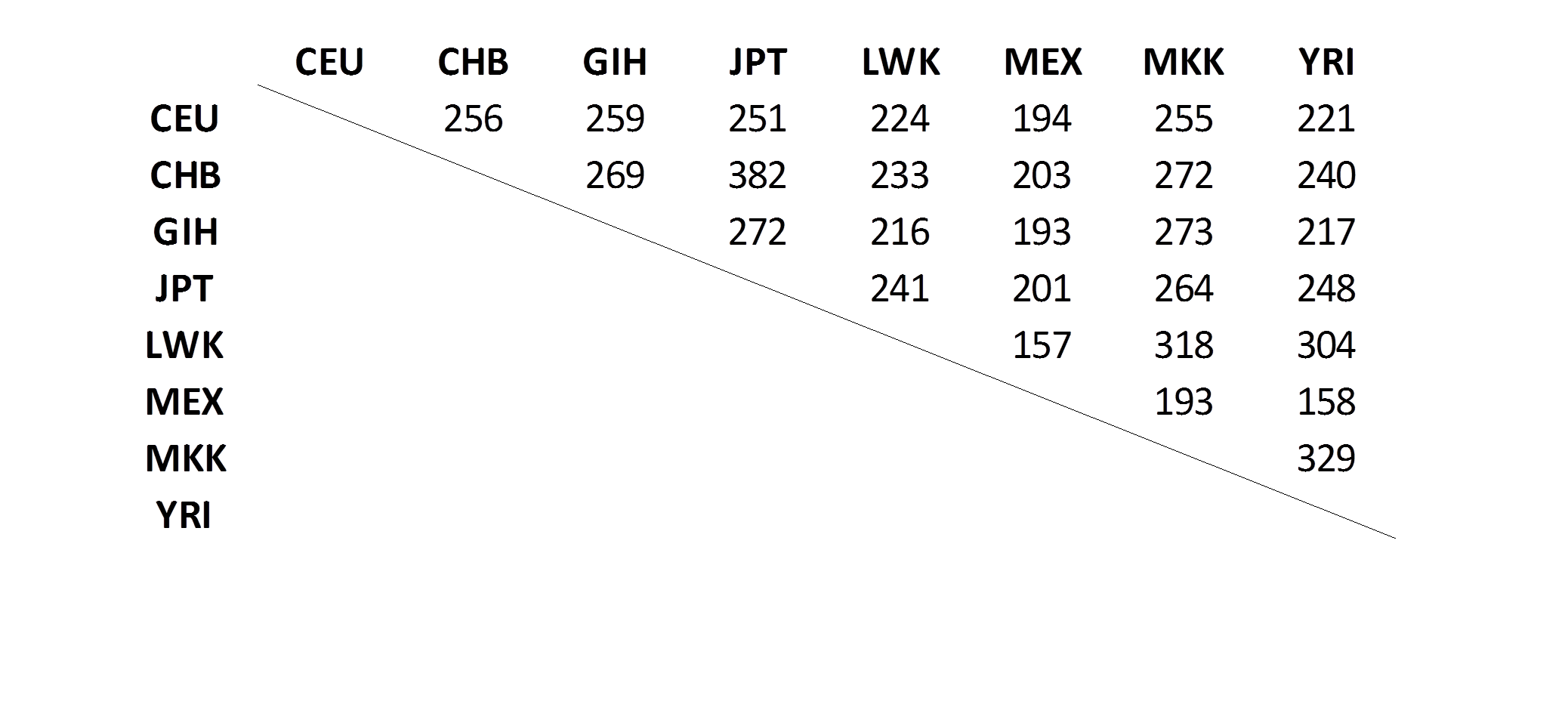

Supplement: Figure S7 — Counts of Ensembl genes with cis- associations significant in pairs of populations. Analysis of associations significant at the 0.01 permutation threshold are shown above the diagonal. Analysis utilized normalized and PCA-corrected expression data. (TIF) [file pgen.1002639.s007.tif]

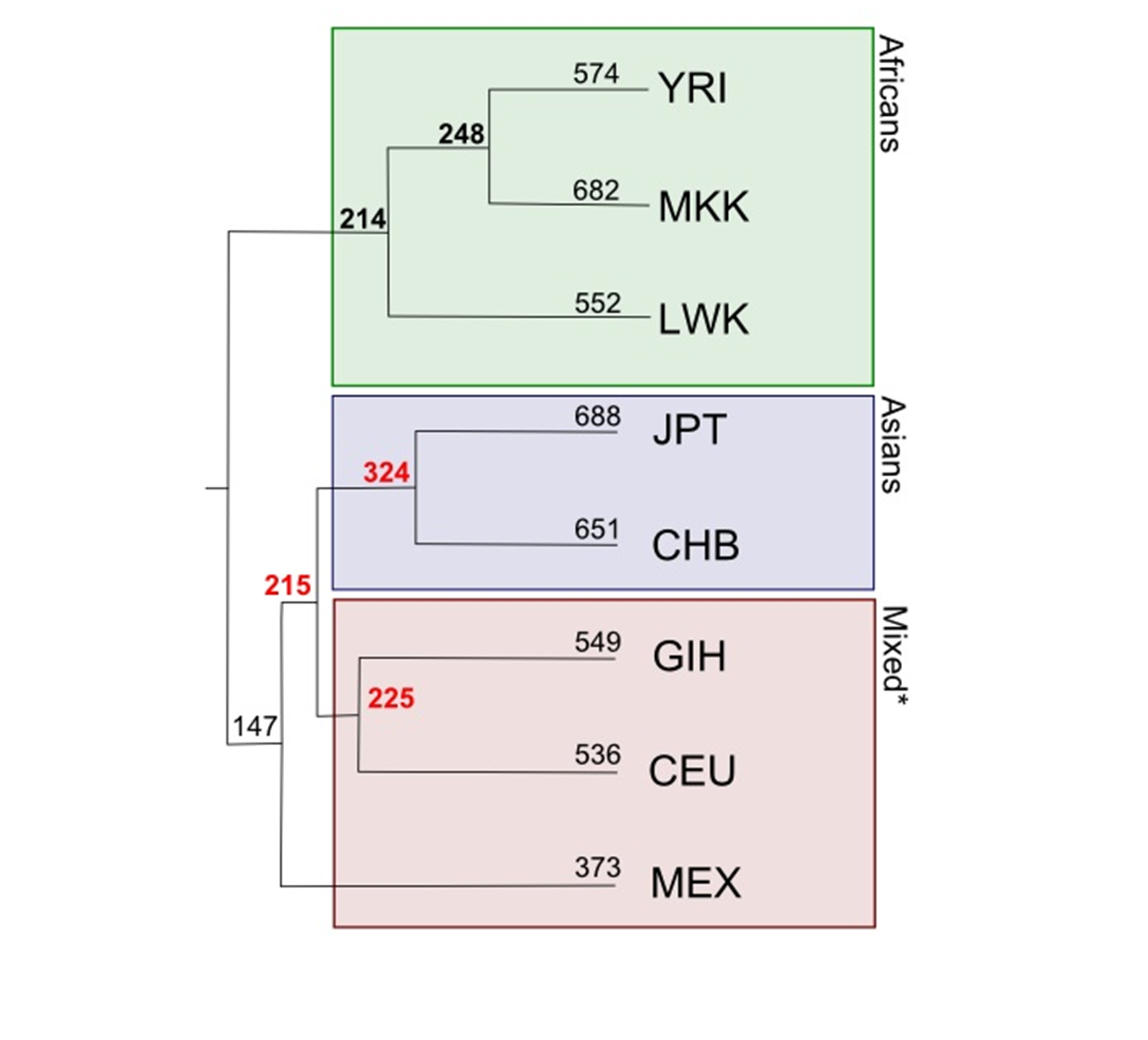

Supplement: Figure S8 — Cis-eQTL sharing across populations using parsimony. Average FST estimates were determined every 10000 SNPs across all pairwise combination of populations and used to create a distance matrix which was assembled into a tree using Neighbour Joining and Matrix Representation with Parsimony. eQTLs were assigned to the tree based on their discovery in at least one population at the 0.01 permutation threshold and then at the 0.1 permutation significance in any additional population. Only one association was selected for each gene; this was done at random. We then created 100 random trees based on reassigning at random the populations at the leaves. Compared to these random trees, the extent of eQTL sharing was increased for some nodes within the observed tree (nodes which have more eQTL sharing than observed in 95% of the random trees are highlighted with red text; this sharing is defined at the same node to respect the overall tree topology, which is maintained, and the respective probability of finding sharing given this topology) . Since this permutation strategy may be overly conservative due to the fact that it does not randomize sharing between populations we also investigated using a different randomization strategy where we randomly assigned the shared populations for each association. Here, we observed not only the previously observed pattern but also observed that the extent of eQTL sharing was increased for nodes within the African subtree (these additional nodes are in black bold text; significance was again defined as excess of sharing compared to 95% of the random trees). These results highlight enrichment of eQTL sharing that are consistent with population structure. (TIF) [file pgen.1002639.s008.tif]

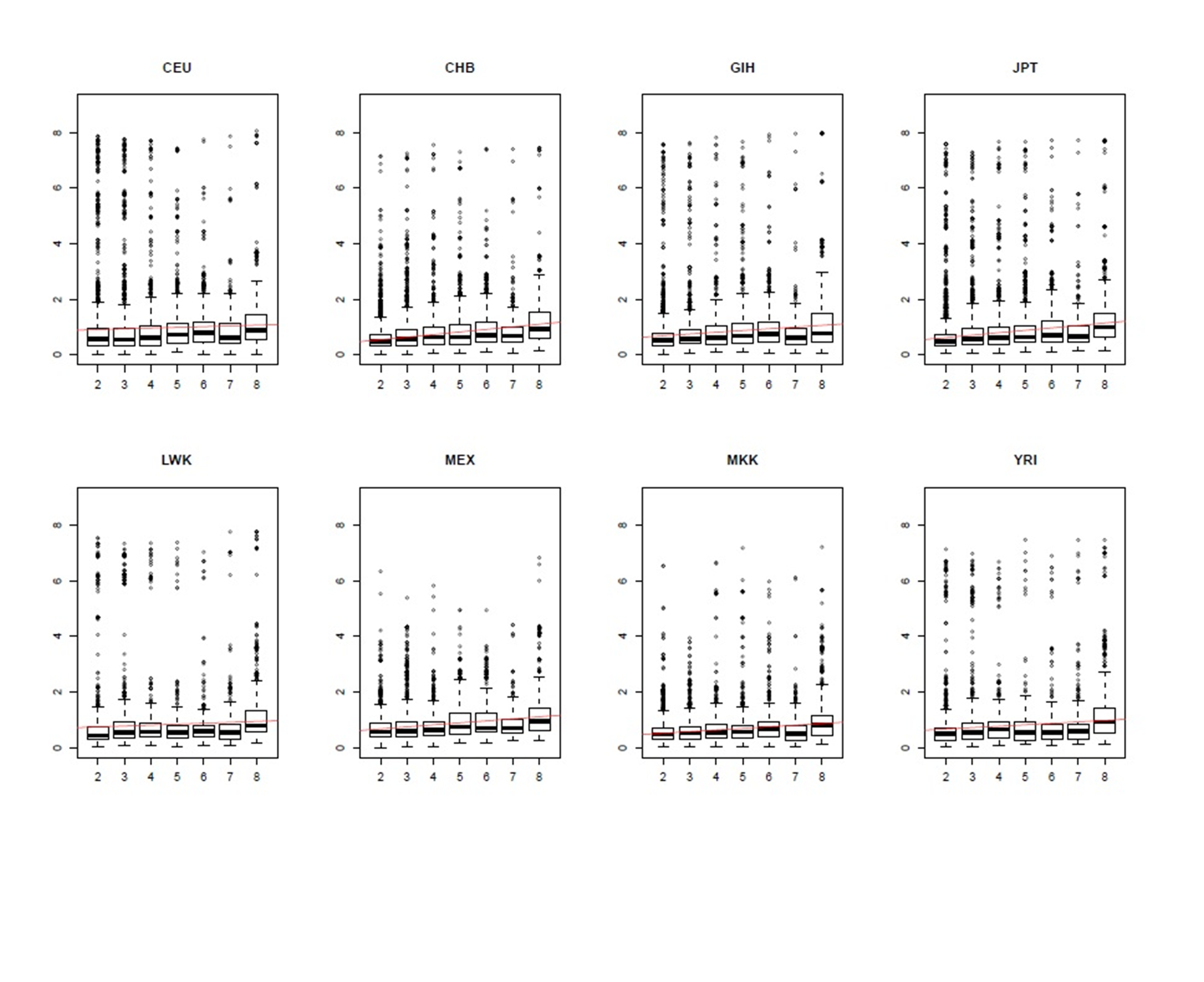

Supplement: Figure S9 — Distribution of effect sizes conditioned on population sharing. We stratified fold change between heterozygotes for 0.1 eQTLs by the number of populations that the eQTL was discovered in at the same threshold. We observed that larger effects are more frequently discovered in multiple populations (Pearson correlation CEU 0.04 p = 1.31e-04, CHB 0.21 p = 2.56e-116, GIH 0.12 p = 1.99e-29, JPT 0.16 p = 3.44e-67, LWK 0.06 p = 7.84e-06, MEX 0.18 p = 6.71e-47, MKK 0.17 p = 1.30e-46, YRI 0.10 p = 2.68e-15). (TIF) [file pgen.1002639.s009.tif]

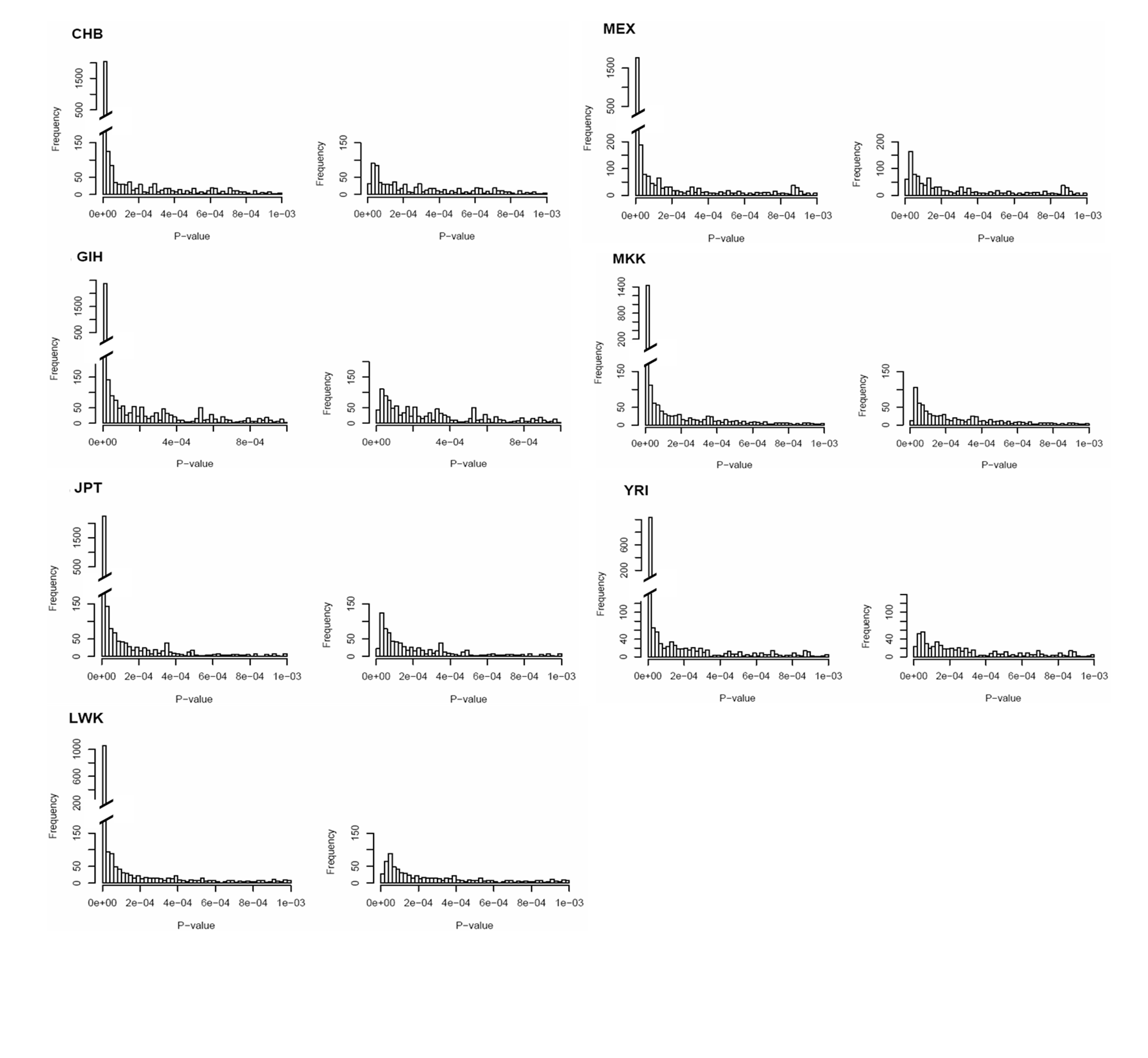

Supplement: Figure S10 — Distribution of corresponding CEU significant (0.01 permutation threshold) cis-association p-values in each of the other seven populations. Left panels display the distribution of all corresponding SNP-probe pair p-values that were significant in CEU, and the right panel shows the distribution of SNP-probe pair p-values after removing those associations that were significant in both CEU and the test population. When the analysis was performed with each other population as the reference, the results remain the same. (TIF) [file pgen.1002639.s010.tif]

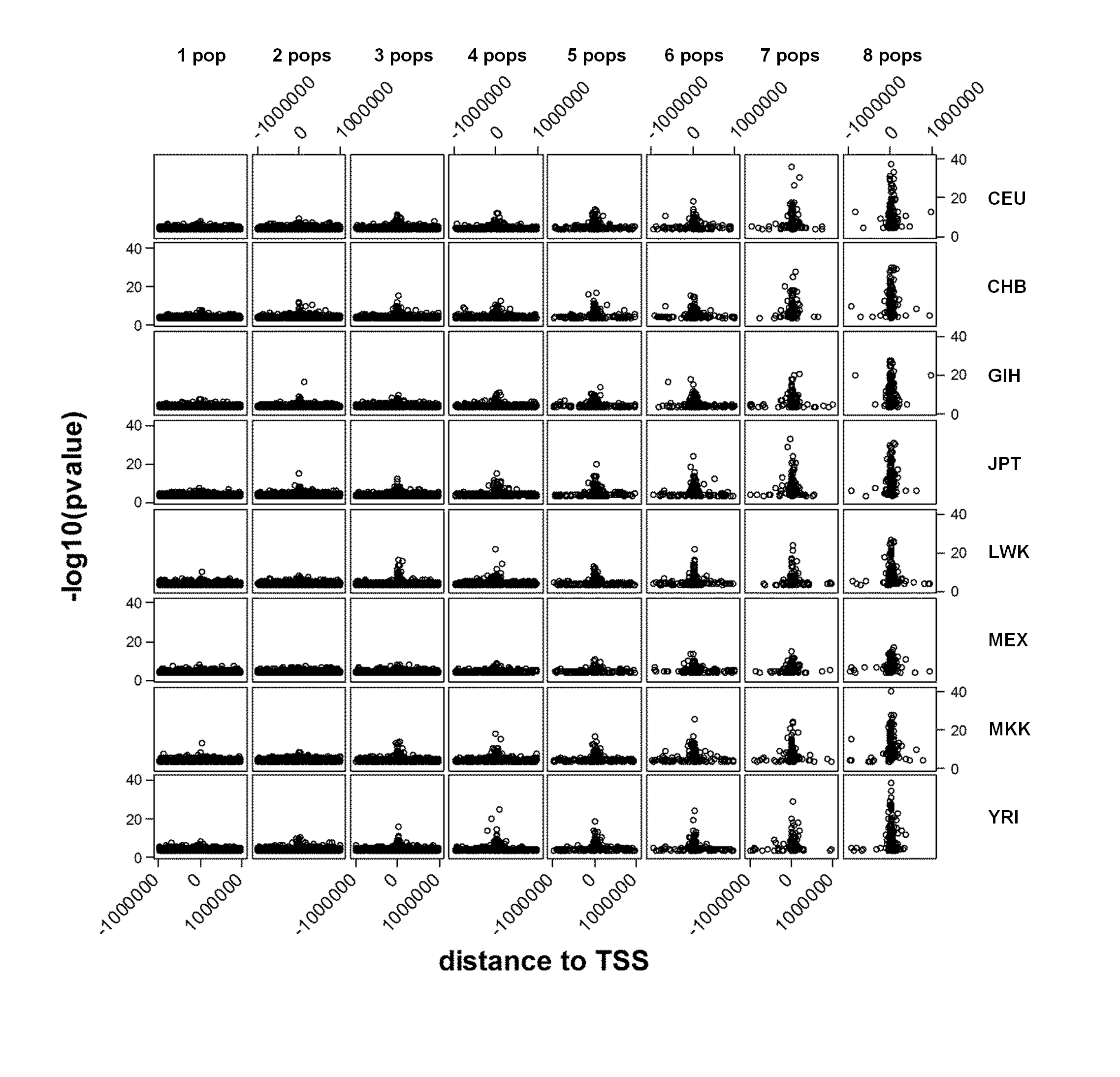

Supplement: Figure S11 — Distribution of cis- associations relative to the transcription start site (TSS) and in relation to population sharing (i.e., whether the gene had a significant association in a single population (1 pop), two populations (2 pops), etc. Shown is −log10 of the p-value plotted against distance from SNP to TSS for each population. Each dot represents the most significant SNP for a significant gene (permutation threshold 0.01) in a population (TIF) [file pgen.1002639.s011.tif]
