# Supplementary material for: Patterns of Cis Regulatory Variation in Diverse Human Populations
Source: PLoS Genet. 2012 Apr 19;8(4):e1002639. doi: 10.1371/journal.pgen.1002639 (PMC3330104; doi:10.1371/journal.pgen.1002639)
Supplement: Table S1 — Summary of the distribution of VST values per probe for all pairwise population comparisons, including the number of genes with VST greater than 0.2. (PDF) [file pgen.1002639.s012.pdf]

Table S1. Summary of the distribution of V_{ST} values per probe for all pairwise population comparisons, including the number of genes with V_{ST} greater than 0.2.

**Population Pair**	**Median**	**Mean**	**Maximum**	**Number of genes V_{ST} > 0.2**
CEU-CHB	0.013	0.040	0.693	893
CEU-GIH	0.012	0.037	0.551	721
CEU-JPT	0.012	0.036	0.614	624
CEU-LWK	0.011	0.034	0.632	591
CEU-MEX	0.011	0.042	0.628	1081
CEU-MKK	0.040	0.079	0.702	2647
CEU-YRI	0.007	0.021	0.456	146
CHB-GIH	0.020	0.056	0.675	1727
CHB-JPT	0.005	0.017	0.238	13
CHB-LWK	0.030	0.079	0.701	3035
CHB-MEX	0.011	0.042	0.504	1155
CHB-MKK	0.054	0.102	0.777	4031
CHB-YRI	0.021	0.044	0.624	806
GIH-JPT	0.017	0.049	0.569	1302
GIH-LWK	0.028	0.075	0.730	2638
GIH-MEX	0.017	0.057	0.634	1955
GIH-MKK	0.029	0.060	0.499	1679
GIH-YRI	0.013	0.035	0.554	578
JPT-LWK	0.025	0.076	0.651	2947
JPT-MEX	0.009	0.034	0.508	535
JPT-MKK	0.034	0.086	0.782	3384
JPT-YRI	0.011	0.035	0.579	623
LWK-MEX	0.021	0.079	0.765	2979
LWK-MKK	0.040	0.090	0.741	3394
LWK-YRI	0.012	0.031	0.399	306
MEX-MKK	0.035	0.093	0.783	3820
MEX-YRI	0.013	0.045	0.576	1196
MKK-YRI	0.021	0.050	0.569	1183
