# Supplementary material for: Patterns of Cis Regulatory Variation in Diverse Human Populations
Source: PLoS Genet. 2012 Apr 19;8(4):e1002639. doi: 10.1371/journal.pgen.1002639 (PMC3330104; doi:10.1371/journal.pgen.1002639)
Supplement: Table S2 — The top 20 most differentiated Gene Ontology functions between all populations. (PDF) [file pgen.1002639.s013.pdf]

Table S2. The top 20 most differentiated Gene Ontology functions between all populations.

GO term	p-value	GO term function
GO:0005634	5.225E-149	nucleus
GO:0005739	4.3975E-91	mitochondrion
GO:0005829	4.8528E-72	cytosol
GO:0005515	1.3043E-66	protein binding
GO:0003723	6.4561E-63	RNA binding
GO:0005737	9.7439E-59	cytoplasm
GO:0000166	6.3562E-53	nucleotide binding
GO:0008380	2.6776E-51	RNA splicing
GO:0005654	8.8023E-50	nucleoplasm
GO:0003676	1.1003E-42	nucleic acid binding
GO:0005730	3.1937E-42	nucleolus
GO:0000398	2.9656E-38	nuclear mRNA splicing, via spliceosome
GO:0051436	9.1724E-38	negative regulation of ubiquitin-protein ligase activity during mitotic cell cycle
GO:0051437	6.0837E-37	positive regulation of ubiquitin-protein ligase activity during mitotic cell cycle
GO:0031145	7.0574E-37	anaphase-promoting complex-dependent proteasomal ubiquitin-dependent protein catabolic process
GO:0016740	2.5797E-33	transferase activity
GO:0005681	4.2676E-33	spliceosome
GO:0005743	4.9103E-33	mitochondrial inner membrane
GO:0005524	5.1422E-32	ATP binding
GO:0006512	8.0575E-30	ubiquitin cycle
