# Supplementary material for: Patterns of Cis Regulatory Variation in Diverse Human Populations
Source: PLoS Genet. 2012 Apr 19;8(4):e1002639. doi: 10.1371/journal.pgen.1002639 (PMC3330104; doi:10.1371/journal.pgen.1002639)
Supplement: Table S3 — For each population, the top 10 most differentiated Gene Ontology functions between the primary population and other populations (and not between any other populations). (PDF) [file pgen.1002639.s014.pdf]

Table S3. For each population, the top 10 most differentiated Gene Ontology functions between the primary population and other populations (and not between any other populations).

	GO term	p-value	GO term function
**CEU**	GO:0006955	6.74E-06	immune response
	GO:0050776	4.05E-05	regulation of immune response
	GO:0000724	2.04E-04	double-strand break repair via homologous recombination
	GO:0030027	6.07E-04	lamellipodium
	GO:0031418	1.17E-03	L-ascorbic acid binding
	GO:0000293	1.40E-03	ferric-chelate reductase activity
	GO:0006968	1.78E-03	cellular defense response
	GO:0000226	2.87E-03	microtubule cytoskeleton organization and biogenesis
	GO:0004114	3.40E-03	3',5'-cyclic-nucleotide phosphodiesterase activity
	GO:0030522	4.20E-03	intracellular receptor-mediated signaling pathway
	GO:0030890	5.02E-03	positive regulation of B cell proliferation
	GO:0005543	5.08E-03	phospholipid binding
**CHB**	GO:0006396	3.88E-06	RNA processing
	GO:0016849	7.67E-04	phosphorus-oxygen lyase activity
	GO:0009190	1.24E-03	cyclic nucleotide biosynthetic process
	GO:0015923	1.76E-03	mannosidase activity
	GO:0009615	1.92E-03	response to virus
	GO:0042826	3.83E-03	histone deacetylase binding
	GO:0006306	3.87E-03	DNA methylation
	GO:0016799	3.94E-03	hydrolase activity, hydrolyzing N-glycosyl compounds
	GO:0006928	6.19E-03	cell motion
	GO:0016018	8.06E-03	cyclosporin A binding
**GIH**	GO:0003779	1.35E-08	actin binding
	GO:0000062	2.14E-06	acyl-CoA binding
	GO:0015629	5.66E-05	actin cytoskeleton
	GO:0046027	1.20E-04	phospholipid:diacylglycerol acyltransferase activity
	GO:0055114	1.91E-03	oxidation reduction
	GO:0005200	2.18E-03	structural constituent of cytoskeleton
	GO:0003841	2.30E-03	1-acylglycerol-3-phosphate O-acyltransferase activity
	GO:0008095	3.20E-03	inositol-1,4,5-triphosphate receptor activity
	GO:0016407	4.27E-03	acetyltransferase activity
	GO:0045103	4.35E-03	intermediate filament-based process
**JPT**	GO:0006520	2.97E-04	amino acid metabolic process
	GO:0006656	2.06E-03	phosphatidylcholine biosynthetic process
	GO:0015020	3.20E-03	glucuronosyltransferase activity
	GO:0030217	5.69E-03	T cell differentiation
	GO:0007089	7.01E-03	traversing start control point of mitotic cell cycle
	GO:0007265	1.19E-02	Ras protein signal transduction
	GO:0006406	1.76E-02	mRNA export from nucleus
	GO:0034100	2.06E-02	L-vinylglycine deaminase activity
	GO:0046688	2.41E-02	response to copper ion
	GO:0006312	2.56E-02	mitotic recombination
	GO:0048496	3.09E-02	maintenance of organ identity
**LWK**	GO:0046872	8.73E-18	metal ion binding
	GO:0006355	2.39E-13	regulation of transcription, DNA-dependent
	GO:0050821	6.85E-08	protein stabilization
	GO:0016568	4.54E-07	chromatin modification
	GO:0005794	9.49E-07	Golgi apparatus
	GO:0006888	1.46E-06	ER to Golgi vesicle-mediated transport
	GO:0045786	1.09E-05	negative regulation of cell cycle
	GO:0003714	3.34E-05	transcription corepressor activity
	GO:0008624	5.13E-05	induction of apoptosis by extracellular signals
	GO:0005694	6.49E-05	chromosome
**MEX**	GO:0005832	2.30E-09	chaperonin-containing T-complex
	GO:0044267	4.22E-09	cellular protein metabolic process
	GO:0006275	1.38E-08	regulation of DNA replication
	GO:0005525	4.60E-08	GTP binding
	GO:0003684	1.29E-06	damaged DNA binding
	GO:0006303	2.02E-06	double-strand break repair via nonhomologous end joining
	GO:0003688	2.57E-06	DNA replication origin binding
	GO:0006164	3.66E-06	purine nucleotide biosynthetic process
	GO:0004019	4.52E-06	adenylosuccinate synthase activity
	GO:0002376	4.52E-06	immune system process
**MKK**	GO:0005743	2.24E-41	mitochondrial inner membrane
	GO:0000502	1.07E-35	proteasome complex
	GO:0006412	8.10E-29	translation
	GO:0005840	4.18E-21	ribosome
	GO:0003735	3.30E-18	structural constituent of ribosome
	GO:0008168	1.93E-17	methyltransferase activity
	GO:0006414	7.99E-16	translational elongation
	GO:0006368	1.49E-12	RNA elongation from RNA polymerase II promoter
	GO:0004527	3.65E-12	exonuclease activity
	GO:0005747	6.12E-12	mitochondrial respiratory chain complex I
**YRI**	GO:0022627	4.74E-08	cytosolic small ribosomal subunit
	GO:0004017	3.41E-03	adenylate kinase activity
	GO:0016791	5.21E-03	phosphatase activity
	GO:0008652	7.10E-03	amino acid biosynthetic process
	GO:0004765	1.30E-02	shikimate kinase activity
	GO:0004725	1.39E-02	protein tyrosine phosphatase activity
	GO:0016311	1.82E-02	dephosphorylation
	GO:0008095	2.04E-02	inositol-1,4,5-triphosphate receptor activity
	GO:0042255	2.10E-02	ribosome assembly
	GO:0006895	2.16E-02	Golgi to endosome transport
