# Supplementary material for: Patterns of Cis Regulatory Variation in Diverse Human Populations
Source: PLoS Genet. 2012 Apr 19;8(4):e1002639. doi: 10.1371/journal.pgen.1002639 (PMC3330104; doi:10.1371/journal.pgen.1002639)
Supplement: Table S4 — Range of Spearman's rho for a) cis- associations detected using ‘REDUCED’ data, and b) Spearman's rho for cis- associations detected using normalized and PCA-corrected data. (PDF) [file pgen.1002639.s015.pdf]

Table S4. Range of Spearman's rho for a) *cis*- associations detected using 'REDUCED' data, and b) Spearman's rho for *cis*- associations detected using normalized and PCA-corrected data.

a)

	Permutation threshold			
	0.01		0.001	
	minimum	maximum	minimum	maximum
**CEU**	0.375	0.903	0.405	0.903
**CHB**	0.438	0.908	0.466	0.908
**GIH**	0.426	0.923	0.464	0.923
**JPT**	0.421	0.92	0.465	0.92
**LWK**	0.438	0.925	0.462	0.925
**MEX**	0.536	0.933	0.603	0.933
**MKK**	0.337	0.896	0.366	0.896
**YRI**	0.372	0.932	0.394	0.932
**average**	0.418	0.918	0.453	0.918

b)

	Permutation threshold			
	0.01		0.001	
	min rho	max rho	min rho	max rho
**CEU**	0.380	0.891	0.411	0.891
**CHB**	0.439	0.908	0.476	0.908
**GIH**	0.434	0.887	0.469	0.887
**JPT**	0.433	0.919	0.458	0.919
**LWK**	0.428	0.878	0.466	0.878
**MEX**	0.572	0.902	0.621	0.902
**MKK**	0.338	0.857	0.358	0.857
**YRI**	0.385	0.897	0.412	0.897
**average**	0.426	0.892	0.459	0.892
