# Supplementary material for: Patterns of Cis Regulatory Variation in Diverse Human Populations
Source: PLoS Genet. 2012 Apr 19;8(4):e1002639. doi: 10.1371/journal.pgen.1002639 (PMC3330104; doi:10.1371/journal.pgen.1002639)
Supplement: Table S5 — Overlap of cis- associated genes that were detected using: 1) normalized and PCA-corrected gene expression data, and 2) ‘REDUCED’ data. (PDF) [file pgen.1002639.s016.pdf]

Table S5. Overlap of *cis*- associated genes that were detected using: 1) normalized and PCA-corrected gene expression data, and 2) 'REDUCED' data.

	permutation threshold									
	0.01					0.001				
	REDUCED[a]	non-RED[b]	overlap[c]	proportion REDUCED[d]	proportion non-RED[e]	REDUCED[a]	non-RED[b]	overlap[c]	proportion REDUCED[d]	proportion non-RED[e]
**CEU**	1966	657	491	0.25	0.75	1253	313	278	0.22	0.89
**CHB**	1950	774	594	0.30	0.77	1218	378	340	0.28	0.90
**GIH**	1984	698	514	0.26	0.74	1219	300	277	0.23	0.92
**JPT**	2131	795	623	0.29	0.78	1327	386	351	0.26	0.91
**LWK**	1794	773	527	0.29	0.68	962	311	265	0.28	0.85
**MEX**	1131	472	305	0.27	0.65	528	165	132	0.25	0.80
**MKK**	2562	947	660	0.26	0.70	1528	411	362	0.24	0.88
**YRI**	2415	799	559	0.23	0.70	1439	328	300	0.21	0.91

[a]Gene counts detected from the 'REDUCED' analysis.

[b]Gene counts detected from the normalized and PCA-corrected analysis.

[c]The intersection of the gene sets from 'REDUCED' and normalized and PCA-corrected analyses.

[d]The proportion of genes that were detected in the 'REDUCED' analysis that were also detected in the normalized and PCA-corrected analysis.

[e]The proportion of genes that were detected in the normalized and PCA-corrected analysis that were also detected in the 'REDUCED' analysis.
