# Supplementary material for: Patterns of Cis Regulatory Variation in Diverse Human Populations
Source: PLoS Genet. 2012 Apr 19;8(4):e1002639. doi: 10.1371/journal.pgen.1002639 (PMC3330104; doi:10.1371/journal.pgen.1002639)
Supplement: Table S6 — Number of Ensembl genes with independent cis-eQTLs at the 0.01 permutation threshold, as determined by stepwise association model. (PDF) [file pgen.1002639.s017.pdf]

Table S6. Number of Ensembl genes with independent *cis*-eQTLs at the 0.01 permutation threshold, as determined by stepwise association model.

**No. of independent *cis*-eQTLs**	**0.01 permutation threshold**							
	**CEU**	**CHB**	**GIH**	**JPT**	**LWK**	**MEX**	**MKK**	**YRI**
**1**	645	765	697	788	772	472	940	786
**2**	10	6	1	6		0	7	11
**3**	2	2						2
**4**		1		1				
**5**					1			
**Total**	657	774	698	795	773	472	947	799
**genes with ≥ 2 *cis*-eQTLs**	12	9	1	7	1	0	7	13
**% genes with ≥ 2 *cis*-eQTLs**	1.83	1.16	0.14	0.88	0.13	0.00	0.74	1.63
