# Supplementary material for: Patterns of Cis Regulatory Variation in Diverse Human Populations
Source: PLoS Genet. 2012 Apr 19;8(4):e1002639. doi: 10.1371/journal.pgen.1002639 (PMC3330104; doi:10.1371/journal.pgen.1002639)
Supplement: Table S7 — Overlap of the most significant cis -eQTL SNP per gene per population (‘REDUCED’ permutation threshold 0.01) with GWAS SNPs from NHGRI GWAS Catalog (Accessed 8/4/2011). (PDF) [file pgen.1002639.s018.pdf]

Table S7. Overlap of the most significant *cis* -eQTL SNP per gene per population ('REDUCED' permutation threshold 0.01) with GWAS SNPs from NHGRI GWAS Catalog (Accessed 8/4/2011)

SNP	Ensembl Gene ID	population	Spearman rho[a]	-log10(pvalue)	SNP distance to TSS (bp)	No.pops with EnsGene *cis* -eQTL	Trait
rs2074518	ENSG00000005156	CEU	0.64	12.9784	16844	4	QT interval
rs470119	ENSG00000025708	CEU	0.46	6.3766	1547	1	Hematological and biochemical traits
rs12188164	ENSG00000063438	CEU	0.54	8.9449	156500	4	Cystic fibrosis severity
rs6537837	ENSG00000065135	CEU	0.75	19.8684	28431	8	Major depressive disorder
rs2290400	ENSG00000073605	CEU	0.51	7.6838	8663	8	Type 1 diabetes
rs6429082	ENSG00000116957	CEU	0.48	6.9352	69401	7	Adiposity
rs1169313	ENSG00000157895	CEU	0.55	9.135	11630	8	Plasma levels of liver enzymes
rs13160562	ENSG00000164307	CEU	0.78	22.7639	32521	8	Alcohol dependence
rs12720356	ENSG00000167807	CEU	0.58	10.3076	-43284	1	Crohn's disease
rs12720356	ENSG00000167807	CEU	0.58	10.3076	-43284	1	Psoriasis
rs694739	ENSG00000168071	CEU	0.52	8.2412	-10462	4	Alopecia areata
rs694739	ENSG00000168071	CEU	0.52	8.2412	-10462	4	Crohn's disease
rs10440635	ENSG00000171522	CEU	0.52	8.0189	-189242	4	Ankylosing spondylitis
rs11978267	ENSG00000185811	CEU	0.51	7.7258	118874	7	Acute lymphoblastic leukemia (childhood)
rs7097	ENSG00000186184	CEU	0.49	7.1281	2533	5	Large B-cell lymphoma
rs2242330	ENSG00000187054	CEU	0.40	4.8707	381895	1	Parkinson's disease
rs9303277	ENSG00000073605	CHB	0.53	6.3094	98434	8	Primary biliary cirrhosis
rs968451	ENSG00000100311	CHB	0.55	6.7767	-29861	3	Primary biliary cirrhosis
rs517811	ENSG00000104490	CHB	0.50	5.4929	192272	2	Cognitive test performance
rs13385191	ENSG00000118961	CHB	0.65	10.2533	134609	7	Prostate cancer
rs3758354	ENSG00000135046	CHB	0.57	7.5415	-2110	4	Schizophrenia, bipolar disorder and depression (combined)
rs2736340	ENSG00000154319	CHB	0.65	10.0553	-11746	8	Rheumatoid arthritis
rs2736340	ENSG00000154319	CHB	0.65	10.0553	-11746	8	Systemic lupus erythematosus
rs13160562	ENSG00000164307	CHB	0.64	9.7479	32521	8	Alcohol dependence
rs181359	ENSG00000185651	CHB	0.75	15.0731	6684	8	Crohn's disease
rs10781499	ENSG00000187796	CHB	0.48	5.1576	1728	3	Ulcerative colitis
rs1008953	ENSG00000198641	CHB	0.53	6.3615	-9851	5	Psoriasis
rs9303277	ENSG00000073605	GIH	0.65	10.324	98434	8	Primary biliary cirrhosis
rs3825932	ENSG00000103811	GIH	0.72	13.3805	1974	8	Type 1 diabetes
rs4580814	ENSG00000113504	GIH	0.47	5.0403	-1094	3	Hematological and biochemical traits
rs13385191	ENSG00000118961	GIH	0.83	21.0682	134609	7	Prostate cancer
rs13160562	ENSG00000164307	GIH	0.78	17.43	32521	8	Alcohol dependence
rs6565681	ENSG00000173821	GIH	0.62	9.148	34768	1	Moyamoya disease
rs1023252	ENSG00000177000	GIH	0.70	12.3663	-32918	6	Natriuretic peptide levels
rs3091242	ENSG00000183726	GIH	0.63	9.5315	9976	6	Erythrocyte sedimentation rate
rs260461	ENSG00000198131	GIH	0.50	5.6934	15555	4	Attention deficit hyperactivity disorder
rs6537837	ENSG00000065135	JPT	0.85	22.807	28431	8	Major depressive disorder
rs7216389	ENSG00000073605	JPT	0.68	11.8324	4954	8	Asthma
rs1550057	ENSG00000109445	JPT	0.54	6.5002	-24667	2	Conduct disorder (case status)
rs4580814	ENSG00000113504	JPT	0.60	8.4605	-1094	3	Hematological and biochemical traits
rs13385191	ENSG00000118961	JPT	0.77	16.2049	134609	7	Prostate cancer
rs13385731	ENSG00000119812	JPT	0.52	6.3516	122476	1	Systemic lupus erythematosus

SNP	Ensembl Gene ID	population	Spearman rho[a]	-log10(pvalue)	SNP distance to TSS (bp)	No.pops with EnsGene *cis*-eQTL	Trait
rs3118470	ENSG00000134453	JPT	0.45	4.6395	-29294	1	Alopecia areata
rs3758354	ENSG00000135046	JPT	0.53	6.5974	-2110	4	Schizophrenia, bipolar disorder and depression (combined)
rs884205	ENSG00000141655	JPT	0.49	5.4204	62337	1	Bone mineral density (spine)
rs4654748	ENSG00000142794	JPT	0.64	9.8485	19438	7	Folate pathway vitamin levels
rs1535	ENSG00000149485	JPT	0.50	5.6697	61034	1	Metabolic syndrome
rs1535	ENSG00000149485	JPT	0.50	5.6697	61034	1	Response to statin therapy
rs1600249	ENSG00000154319	JPT	0.69	12.1534	-27411	8	Rheumatoid arthritis
rs17021918	ENSG00000163110	JPT	0.49	5.539	189839	5	Prostate cancer
rs13160562	ENSG00000164307	JPT	0.58	7.8472	32521	8	Alcohol dependence
rs12338076	ENSG00000165661	JPT	0.51	5.8758	15947	5	Height
rs7216389	ENSG00000172057	JPT	0.77	16.8491	13905	8	Asthma
rs3091242	ENSG00000183726	JPT	0.68	11.7761	9976	6	Erythrocyte sedimentation rate
rs4821112	ENSG00000185651	JPT	0.72	13.4934	42804	8	Hematological and biochemical traits
rs2860580	ENSG00000196306	JPT	0.57	7.6414	51302	8	Nasopharyngeal carcinoma
rs260461	ENSG00000198131	JPT	0.58	7.853	15555	4	Attention deficit hyperactivity disorder
rs3825932	ENSG00000103811	LWK	0.71	13.2979	1974	8	Type 1 diabetes
rs281379	ENSG00000126453	LWK	0.51	5.9256	-954125	1	Crohn's disease
rs2273	ENSG00000138744	LWK	0.53	6.4292	-27222	8	Longevity
rs1048886	ENSG00000154079	LWK	0.58	8.0156	12569	6	Type 2 diabetes
rs2736340	ENSG00000154319	LWK	0.64	10.0644	-11746	8	Rheumatoid arthritis
rs2736340	ENSG00000154319	LWK	0.64	10.0644	-11746	8	Systemic lupus erythematosus
rs12029454	ENSG00000158850	LWK	0.51	5.9764	-985803	1	QT interval
rs13160562	ENSG00000164307	LWK	0.46	4.7454	32521	8	Alcohol dependence
rs9822268	ENSG00000185614	LWK	0.60	8.3886	-120958	1	Ulcerative colitis
rs12680655	ENSG00000066827	MEX	0.64	5.727	87944	6	Height
rs9303277	ENSG00000073605	MEX	0.59	4.7249	98434	8	Primary biliary cirrhosis
rs5751901	ENSG00000100031	MEX	0.63	5.3691	12548	5	Protein quantitative trait loci
rs1260326	ENSG00000115241	MEX	0.70	6.9657	-98444	3	C-reactive protein
rs1260326	ENSG00000115241	MEX	0.70	6.9657	-98444	3	Chronic kidney disease
rs1260326	ENSG00000115241	MEX	0.70	6.9657	-98444	3	Hematological and biochemical traits
rs1260326	ENSG00000115241	MEX	0.70	6.9657	-98444	3	Hypertriglyceridemia
rs1260326	ENSG00000115241	MEX	0.70	6.9657	-98444	3	Other metabolic traits
rs1260326	ENSG00000115241	MEX	0.70	6.9657	-98444	3	Triglycerides
rs1260326	ENSG00000115241	MEX	0.70	6.9657	-98444	3	Two-hour glucose challenge
rs1260326	ENSG00000115241	MEX	0.70	6.9657	-98444	3	Waist circumference and related phenotypes
rs13385191	ENSG00000118961	MEX	0.77	9.0918	134609	7	Prostate cancer
rs4654748	ENSG00000142794	MEX	0.67	6.4068	19438	7	Folate pathway vitamin levels
rs2618476	ENSG00000154319	MEX	0.82	10.4371	-20314	8	Systemic lupus erythematosus
rs13160562	ENSG00000164307	MEX	0.70	7.1185	32521	8	Alcohol dependence
rs255049	ENSG00000167264	MEX	0.60	4.9422	-43712	1	HDL cholesterol
rs6049839	ENSG00000185019	MEX	0.60	4.9054	621975	3	Systemic lupus erythematosus
rs7678436	ENSG00000109805	MKK	0.39	5.5855	-14559	2	Height
rs6918981	ENSG00000112664	MKK	0.38	5.3604	121927	2	Height
rs13385191	ENSG00000118961	MKK	0.55	11.3519	134609	7	Prostate cancer

SNP	Ensembl Gene ID	population	Spearman rho[a]	-log10(pvalue)	SNP distance to TSS (bp)	No.pops with EnsGene *cis*-eQTL	Trait
rs3758354	ENSG00000135046	MKK	0.38	5.3503	-2110	4	Schizophrenia, bipolar disorder and depression (combined)
rs2277027	ENSG00000135074	MKK	0.44	7.208	70392	6	Pulmonary function
rs755109	ENSG00000136932	MKK	0.54	10.9106	-11351	3	Quantitative traits
rs735396	ENSG00000157895	MKK	0.48	8.5433	15456	8	N-glycan levels
rs10440635	ENSG00000171522	MKK	0.39	5.5665	-189242	4	Ankylosing spondylitis
rs8049439	ENSG00000178952	MKK	0.44	7.0041	20128	5	Inflammatory bowel disease (early onset)
rs7586970	ENSG00000064989	YRI	0.40	4.6838	-30482	1	Coronary heart disease
rs1412444	ENSG00000107798	YRI	0.46	6.2154	171387	4	Coronary heart disease
rs3729639	ENSG00000125122	YRI	0.46	6.1631	35400	2	Coronary heart disease
rs3758354	ENSG00000135046	YRI	0.44	5.4498	-2110	4	Schizophrenia, bipolar disorder and depression (combined)
rs2736340	ENSG00000154319	YRI	0.53	8.3462	-11746	8	Rheumatoid arthritis
rs2736340	ENSG00000154319	YRI	0.53	8.3462	-11746	8	Systemic lupus erythematosus
rs13160562	ENSG00000164307	YRI	0.66	14.0736	32521	8	Alcohol dependence
rs17119280	ENSG00000172456	YRI	0.53	8.4766	36421	1	Response to antipsychotic therapy (extrapyramidal side effects
rs8049439	ENSG00000178952	YRI	0.58	10.461	20128	5	Inflammatory bowel disease (early onset)

[a]Absolute value of Spearman's rho
